# Variable habitat use supports fine-scale population differentiation of a freshwater piscivore (northern pike, *Esox lucius*) along salinity gradients in brackish lagoons

**DOI:** 10.1007/s00442-024-05627-7

**Published:** 2024-10-18

**Authors:** Timo D. Rittweg, Clive Trueman, Michael Wiedenbeck, Jan Fietzke, Christian Wolter, Lauren Talluto, Stefan Dennenmoser, Arne Nolte, Robert Arlinghaus

**Affiliations:** 1 Leibniz Institute of Freshwater Ecology and Inland Fisheries (IGB), Müggelseedamm 310, 12587 Berlin, Berlin, Germany; 2 School of Ocean and Earth Science, University of Southampton Waterfront Campus, European Way, Southampton, SO143ZH UK; 3 German Research Center for Geosciences (GFZ) Potsdam, Telegrafenberg, 14473 Potsdam, Brandenburg Germany; 4 GEOMAR Helmholtz Center for Ocean Research Kiel, Wischhofstr. 1-3, 24148 Kiel, Schleswig-Holstein Germany; 5 Research Group Fluvial Ecosystem Ecology, Department of Ecology, University of Innsbruck, Technikerstr. 25, 6020 Innsbruck, Austria; 6 Working Group Ecological Genomics, Institute of Biology and Environmental Sciences, Carl Von Ossietzky Universität Oldenburg, Carl Von Ossietzky-Str. 9-11, 26111 Oldenburg, Germany; 7 Division of Integrative Fisheries Management, Faculty of Life Sciences, Humboldt-Universität zu Berlin, Unter den Linden 6, 10099 Berlin, Germany

**Keywords:** Ecological niche, Evolution, Life histories, Growth, Partial migration

## Abstract

In mobile animals, selection pressures resulting from spatio-temporally varying ecological factors often drive adaptations in migration behavior and associated physiological phenotypes. These adaptations may manifest in ecologically and genetically distinct ecotypes within populations. We studied a meta-population of northern pike (*Esox lucius*) in brackish environments and examined intrapopulation divergence along environmental gradients. Behavioral phenotypes in habitat use were characterized via otolith microchemistry in 120 individuals sampled from brackish lagoons and adjacent freshwater tributaries. We genotyped 1514 individual pike at 33 highly informative genetic markers. The relationship between behavioral phenotype and genotype was examined in a subset of 101 pikes for which both phenotypic and genomic data were available. Thermosaline differences between juvenile and adult life stages indicated ontogenetic shifts from warm, low-saline early habitats towards colder, higher-saline adult habitats. Four behavioral phenotypes were found: Freshwater residents, anadromous, brackish residents, and cross-habitat individuals, the latter showing intermediary habitat use between brackish and freshwater areas. Underlying the behavioral phenotypes were four genotypes, putative freshwater, putative anadromous, and two putatively brackish genotypes. Through phenotype-genotype matching, three ecotypes were identified: (i) a brackish resident ecotype, (ii) a freshwater ecotype expressing freshwater residency or anadromy, and (iii) a previously undescribed intermediary cross-habitat ecotype adapted to intermediate salinities, showing limited reliance on freshwater. Life-time growth of all ecotypes was similar, suggesting comparable fitness. By combining genetic data with lifelong habitat use and growth as a fitness surrogate, our study revealed strong differentiation in response to abiotic environmental gradients, primarily salinity, indicating ecotype diversity in coastal northern pike is higher than previously believed.

**Graphical abstract:**

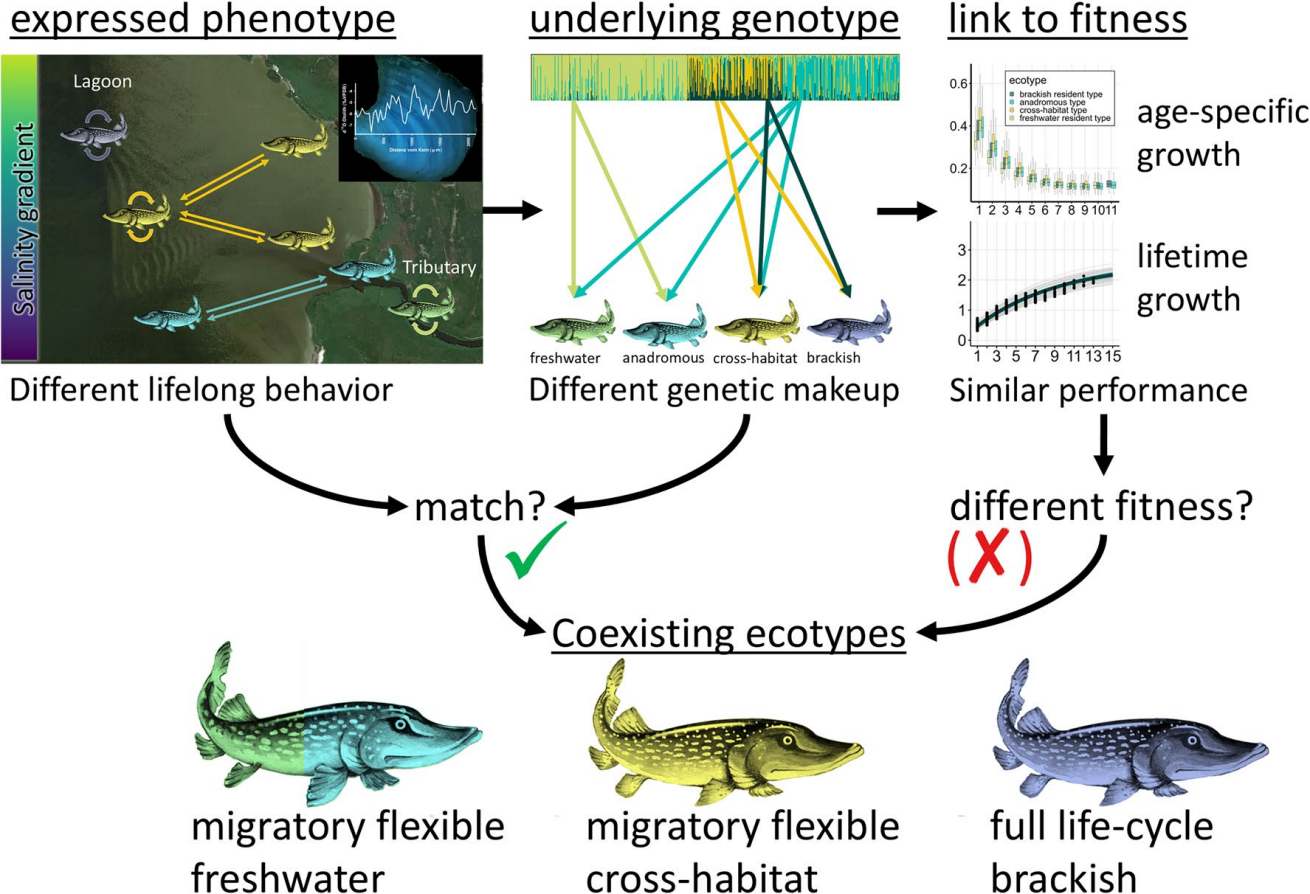

**Supplementary Information:**

The online version contains supplementary material available at 10.1007/s00442-024-05627-7.

## Introduction

Ecological factors like food availability, predation, and abiotic environmental conditions shape niche spaces and the fitness landscape of organisms in the wild (Roff 2002). Selection pressures vary over time and space due to density fluctuations, environmental gradients, and environmental stochasticity (Bell 2010). Organisms adapt to fluctuating selection through various traits and processes, e.g., behavioral shifts in habitat use, migration, physiological adaptation to local environmental factors, or microevolutionary changes in adaptive life history traits (Felmy et al. 2022; Sunde et al. 2022; Tibblin et al. 2015, 2016). Trade-offs between traits and limited ability to generalize (Rosenzweig 1974) cause intraspecific phenotypic and genetic variability, fostering ecotype evolution (Brown 1990) and sympatric speciation (Doebeli and Dieckmann 2003). Although terminology varies (Clemens and Schreck 2021), ecotypes are characterized by both phenotypic (e.g., in morphology, physiology, and behavior) and genetic differentiation (Stronen et al. 2022). Documenting ecotypes therefore requires that phenotypic and genetic data are combined, which is rarely the case (Clemens and Schreck 2021; Stronen et al. 2022). For example, out of 112 publications reviewed by Stronen et al. (2022) that use the term ecotype, only 53% incorporated genetic analyses, which was attributed to limited availability of genomic resources, particularly for nonmodel organisms.

Environmental conditions near the edge of a species tolerance can prompt local evolutionary adaptations and cause population diversification (Pörtner et al. 2010). Brackish estuarine systems pose such challenges, particularly for osmoregulating ectotherms, such as fishes, where salinity and temperature are key ecological factors (Kültz 2015; Magnuson et al. 1979). Spatio-temporal variation in these factors invokes trade-offs among traits and metabolic costs on the individual level (Sokolova 2021), driving adaptive responses, such as the evolution of variable migration strategies (Delgado and Ruzzante 2020). A possible outcome is partial migration, when varying migration behaviors are expressed along a behavioral continuum (Chapman et al. 2011), and behavioral endpoints (such as residency in one habitat) often correlate with the extremes of underlying environmental factors (Cagnacci et al. 2011). Variable migration behaviors, along with genetic differences, have been described in several coastal fish species, indicating ecotype evolution (e.g., Nordahl et al. 2019; Dennenmoser et al. 2017; Kusakabe et al. 2017). Adaptations to environmental factors also occur in less mobile life stages, such as eggs or larvae, as physiological tolerances are often size-specific in fishes (Werner 1988). Larger individuals often exhibit greater osmoregulatory capacity and lower temperature preference (Lindmark et al. 2022; Varsamos et al. 2005). Thus, mobile species in brackish environments can also be expected to adapt behaviorally through ontogenetic habitat shifts, e.g., favoring warmer, less saline juvenile habitats and colder, more saline adult habitats (Casselman and Lewis 1996; Pursiainen et al. 2021).

Genetically, intrapopulation diversification can arise from isolation by environment (IBE), where individuals become reproductively isolated through adaptation to local environmental factors (Wang and Bradburd 2014), and isolation by distance (IBD), where geographic distance limits gene flow (Wright 1943). Additionally, reproductive timing (isolation by time, IBT, Hendry and Day 2005), and natural or anthropogenic barriers (isolation by resistance, IBR, McRae 2006) can limit gene flow among subpopulations. The northern pike (*Esox lucius*), a mesothermal, stenohaline freshwater predator (Jacobsen and Engström-Öst 2018), presents a suitable model to study intrapopulation diversification (Forsman et al. 2015), as it exhibits all these isolation mechanisms and multiple phenotypes across its distribution range in the subarctic northern hemisphere (Bekkevold et al. 2015; Eschbach et al. 2021; Nordahl et al. 2019; Sunde et al. 2022; Tibblin et al. 2016). Pike are strongly phytophilic, relying on macrophytes both for reproduction as well as for foraging and predator avoidance (Grimm 1981). They exhibit limited mobility and dispersal (Dhellemmes et al. 2023a) and show natal homing (Engstedt et al. 2014; Miller et al. 2001; Tibblin et al. 2016). Pike have colonized brackish habitats in the Baltic Sea from glacial freshwater refuges (Maes et al. 2003), inhabiting brackish water up to 15 Practical Salinity Units (PSU) (Jacobsen and Engström-Öst 2018). Previous studies indicated weak genetic differentiation between coastal populations, but large-scale IBD patterns, most likely explained by limited dispersal, as pike prefer shallow vegetated habitats (Laikre et al. 2005; Maes et al. 2003; Wennerström et al. 2017). More recent research, however, identified genetic differentiation at small geographic scales in coastal pike populations (Diaz-Suarez et al. 2022; Möller et al. 2020; Nordahl et al. 2019; Wąs-Barcz et al. 2023). Although IBD patterns were also present on a local scale (e.g., Möller et al. 2020), several studies found strong evidence for IBE through local adaptation (Sunde et al. 2018, 2019, 2022). Key abiotic factors driving fine-scale adaptive population differentiation in pike include salinity (Jørgensen et al. 2010; Sunde et al. 2018, 2022; Arlinghaus et al. 2023), and local temperature (Sunde et al. 2019). Subpopulation-specific variation in early life history traits, growth rates, vertebra number and reproductive investment (Berggren et al. 2016; Tibblin et al. 2015, 2016) indicated the evolution of ecotypes with limited gene flow.

The literature on coastal pike often emphasizes two ecotypes: A brackish resident, adapted to reproduce in salinities up to 10 PSU (Arlinghaus et al. 2023; Jørgensen et al. 2010; Sunde et al. 2018), and an anadromous ecotype that forages in coastal sites but returns to freshwater for reproduction (Arlinghaus et al. 2023; Larsson et al. 2015; Müller et al. 1986). This dichotomy mirrors ecotype literature in various fish species, such as benthic vs. pelagic (e.g., Blain et al. 2023), limnic vs. marine (e.g., Kusakabe et al. 2017) or migratory vs. resident (e.g., Olsson et al. 2006). However, intermediary phenotypes with flexible habitat use between freshwater and brackish water have repeatedly been reported in coastal fishes (Almeida et al. 2023; Kerr et al. 2007, 2009; Limburg et al. 2001; Rohtla et al. 2020, 2023; Russell et al. 2022), challenging the dichotomous perspective and hinting at patterns of partial migration (Chapman et al. 2011). The presence of additional phenotypes has also been proposed in coastal pike populations (such as freshwater residents in tributaries, Birnie-Gauvin et al. 2019), but without genetic evidence to confirm them as ecotypes.

Previous studies on habitat use of coastal pike often focused on specific habitats (coastal habitats only in Engstedt et al. 2010; Jacobsen et al. 2017; or freshwater tributaries only in Engstedt et al. 2014; Tibblin et al. 2015), or specific life stages (natal origin, Möller et al. 2019, or adult movements, Dhellemmes et al. 2023a). Therefore, much of this past research only resolved short periods of individual life cycles, and only for subsets of coastal populations. High-resolution otolith microchemistry offers a powerful complementary tool to purely genetic studies (Trueman et al. 2012) by retrospectively identifying individual-level movements between freshwater and brackish habitats throughout their entire lives, for example through strontium to calcium ratios (Sr:Ca, Kafemann et al. 2000), and by reconstructing thermal environments experienced by individuals through oxygen isotope ratios (δ^18^O values, Patterson et al. 1993). Lifelong individual assessments that cover all possible phenotypes, and link habitat use to genetic diversity and fitness surrogates, may reveal crucial aspects of the species’ evolutionary history (Durif et al. 2023), and aid in detecting additional ecotypes (Stronen et al. 2022).

The study objective was to identify the full suite of behavioral phenotypes and genotypes present in a coastal pike population along a salinity gradient from freshwater tributaries to mesohaline lagoons, compare subpopulation-level fitness (using growth as a proxy), and identify ecotypes. To assess evolutionary divergence, individual-level thermosaline habitat use was matched to individual-level genotypic information, using genomic markers involved in adaptive divergence along a salinity gradient. We hypothesized that (1) thermosaline habitat use changes with size and age, with smaller pike inhabiting warmer, less saline habitats that become increasingly colder and more saline as individuals grow; (2) pike in brackish lagoons and their tributaries have evolutionarily diverged into multiple phenotypically and genetically distinct ecotypes; and (3) adaptation to salinity is a driver of ecotype evolution of pike in coastal brackish lagoons and adjacent tributaries.

## Materials and methods

### Study site and sampling

**Fig. 1:**
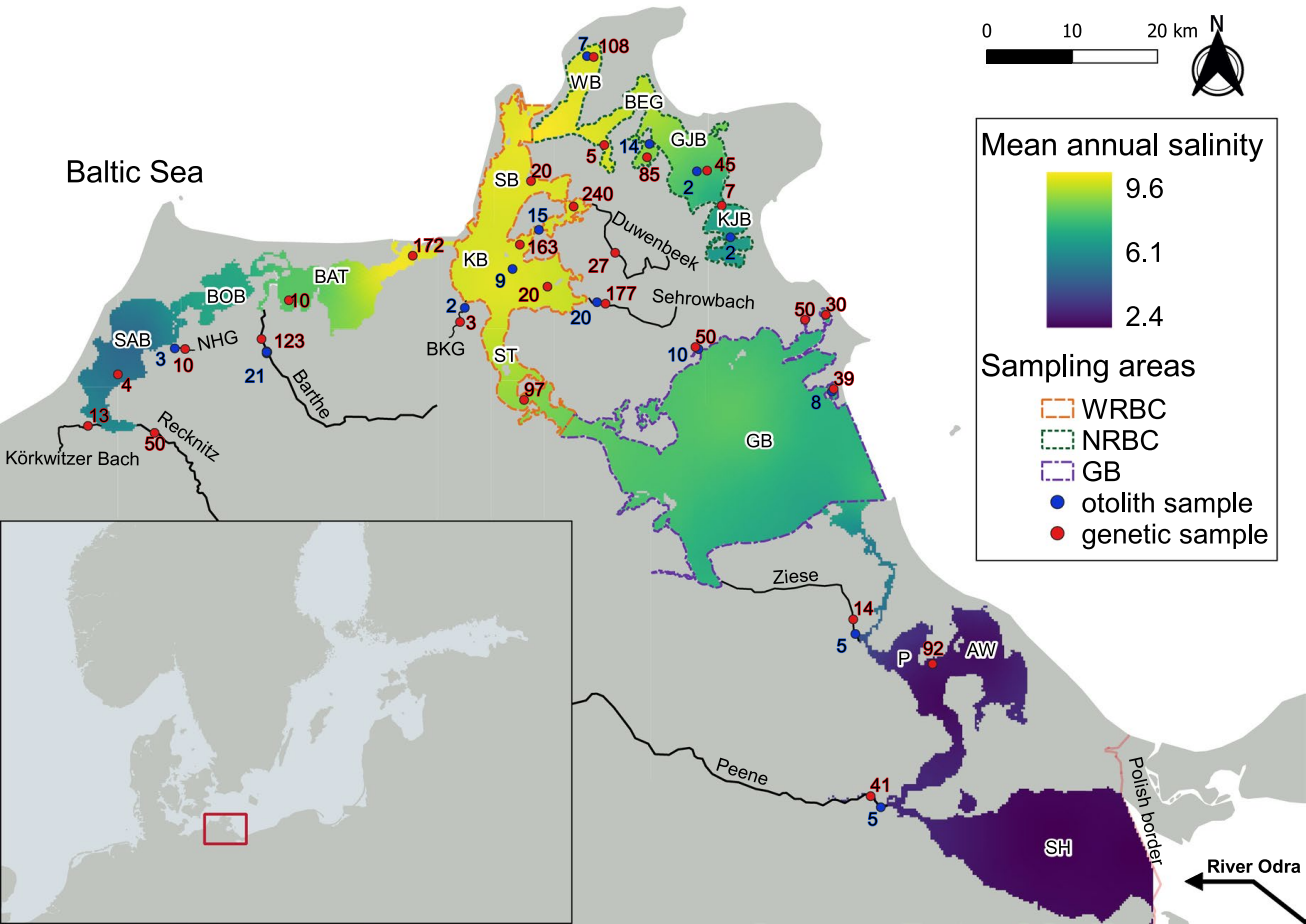
Capture locations of northern pike (*Esox lucius*) between July 2019 and April 2022 around Rügen island, Germany. Numbers highlighted in blue indicate the number of pike captured for otolith microchemistry (total *N* = 120) at the respective locations, numbers highlighted in red indicate the total number of genetic samples (total *N* = 1514) from a location. Major brackish lagoon chains used for otolith sampling are outlined in color: *WRBC* Western Rügen Bodden Chain, *NRBC* Northern Rügen Bodden Chain, *GB* Greifswalder Bodden. Labels with white text buffer are referring to brackish lagoons, labels without text buffer to freshwater tributaries. Single lagoon abbreviations (from west to east): *SAB* Saaler Bodden, *BOB* Bodstedter Bodden, *BAT* Barther Bodden & Grabow, *KB* Kubitzer Bodden, *SB* Schaproder Bodden, *ST* Strelasund, *WB* Wieker Bodden, *BEG* Breeger Bodden, *GJB* Großer Jasmunder Bodden, *KJB* Kleiner Jasmunder Bodden, *P* Peenestrom, *AW* Achterwasser, *SH* Stettiner Haff. Tributary abbreviations: *NHG* Neuendorfer Hechtgraben, *BKG* Badendycksgraben

We studied the pike population in brackish lagoons surrounding Rügen island in the southern Baltic Sea, Germany. This interconnected system of lagoons and tributaries features strong environmental gradients in salinity and temperature. Freshwater from rivers (e.g., Recknitz, Barthe, Odra, Peene) mixes with brackish water from the Baltic Sea, forming oligohaline lagoons towards the west (Saaler Bodden, SAB, Bodstedter Bodden, BOB, Fig. 1) and southeast (Peenestrom, P, Achterwasser, AW, and Stettiner Haff, SH, Fig. 1). Additionally, numerous smaller creeks and drainage ditches, many of which were obstructed by pump sheds and shutters during melioration measures in the 1970s (Roser et al. 2023), drain into the lagoons (Fig. 1). Salinity levels increase from an annual average of 3–5 PSU in the western oligohaline lagoons to 8–10 PSU in the northwestern mesohaline lagoons (Western Rügen Bodden chain, WRBC, and Northern Rügen Bodden chain, NRBC, Fig. 1), and from 2 to 3 PSU in the southeastern oligohaline lagoons to 6.5 PSU in the northeastern mesohaline lagoon (Greifswalder Bodden, GB, Fig. 1). A temperature gradient spans from warmer average annual temperatures in the eutrophic lagoons in the southwest (SAB, BOB) and southeast (P), towards colder, mesotrophic lagoons in the north (WRBC and NRBC) (Fig. S1, Table S1).

To assess the full range of phenotypic diversity along the salinity and temperature gradient, we sampled 66 adult pike (> 95% maturation size, Palder et al. 2023) (43 females, 23 males, 40 – 126 cm total length, mean ± standard deviation, 82 ± 17 cm, 1 – 13 years in age) from the three major mesohaline lagoon chains WRBC (N = 24), NRBC (N = 24) and GB (N = 18), and 54 adult pike (36 females, 18 males, 42 – 106 cm, mean total length 77 ± 13 cm, 1 – 11 years in age) from adjacent freshwater tributaries (Fig. 1) over three consecutive years (2019 – 2022) for otolith microchemistry. To capture both resident and migratory phenotypes, we collected fish outside the spawning season (outside March and April) in the brackish lagoons, and during the spawning season (March and April) in the freshwater tributaries, when we assumed all phenotypes were present in a given habitat (Table S2). To ensure salinity differences between habitats were sufficient to be measured via otolith microchemistry, we excluded oligohaline lagoons. To sample the full range of freshwater residents, a small additional set of fish (*N* = 11) was captured in tributaries outside the spawning season in July 2019. To cover variability across larger spatial scales and different habitat types, tributaries of varying size and location were sampled: Larger rivers Barthe (*N* = 23) and Peene (*N* = 5), medium-sized creeks Sehrowbach (*N* = 20) and Ziese (*N* = 5), and two small drainage ditches, Neuendorfer Hechtgraben (NHG, *N* = 3) and Badendycksgraben (BKG, *N* = 2, Fig. 1). To mitigate gear-induced bias on size and age structure, multiple gears were employed (Wilson et al. 2015): Gill nets, fyke nets and angling in brackish lagoons, as well as electrofishing in freshwater tributaries (Table S2). Fish were sampled by the research team and by contracted fishers, measured (total length, mm), internally sex determined, fin clipped for genetic analyses, and sagittal otoliths were retrieved. To represent different age classes for lifelong phenotypic assessments, pike were selected randomly in a length-stratified manner. We aimed for an equal sex ratio at 5 cm size class intervals and equal sample sizes for each lagoon chain and tributary. Sampling limitations and sex-dimorphic growth (Casselman 1995) did ultimately not allow for an equal sex ratio (Figs. S2, S3). Low capture rates did not allow for length-stratified sampling in all tributaries, so tributary samples were pooled for some analyses (Table S2). However, we acquired length-stratified samples in two tributaries draining into two different lagoons (Sehrowbach and Barthe, Table S2), and supplemented samples from the other tributaries to cover the spatial gradient of freshwater tributaries in the region (Fig. 1).

To assess genetic diversity present in the lagoon system at the individual level, we sampled 1514 individuals for which fin clips were collected non-lethally via cooperating fishers and anglers and by the research team across the entire salinity gradient (including oligohaline lagoons) and all major tributaries (Fig. 1). In addition, 6 fish were collected from a freshwater lake (Kleiner Döllnsee) roughly 250 km to the south of the study system. All fish were individually genotyped (see below). For a subset of pike where both otolith microchemistry and genotype information was available (*N* = 101), data were used for phenotype-genotype matching as described below.

### Otolith microchemistry

To assess the lifelong thermal environment experienced by each individual fish, intraotolith δ^18^O (‰ relative to Vienna Pee Dee Belemnite, VPDB) values were determined at 35 µm intervals along transects from the otolith core to the outer edge, covering all visible year rings (annuli) on transverse thin sections of sagittal otoliths with secondary ion mass spectrometry (SIMS) at the GeoForschungsZentrum (GFZ), Potsdam. To resolve the lifelong saline environment, Sr:Ca ratios (mg/g) were determined at GEOMAR Helmholtz Centre for Ocean Research Kiel with laser ablation inductively coupled plasma mass spectrometry (LA-ICPMS), at 5.5 µm intervals within the same core–edge transects used for SIMS. An average of 40 combined elemental determinations per individual otolith equated to an average spatial resolution of 14 values per annulus, achieving sub-monthly temporal resolution. To correct δ^18^O values for salinity-induced effects (Darnaude et al. 2014), we extracted residuals from a linear regression of δ^18^O on Sr:Ca values from the same location on the otolith. δ^18^O residuals were then assumed to reflect lifelong individual variation in the thermal environment. Age, annual otolith growth increments and radius of each otolith were determined to estimate the growth rate as per Rittweg et al. (2024). To avoid back-calculation assumptions, growth analyses were conducted on otolith annual increments (supplement, section B).

### Analysis of otolith transects

We used individual-level salinity and thermal metrics to identify habitat use patterns in pike. We applied dynamic time warp (DTW) clustering on individual elemental transects of Sr:Ca and δ^18^O residuals, pooling samples from brackish lagoons (*N* = 66) and tributaries (*N* = 54). We performed an agglomerative hierarchical clustering following Hegg and Kennedy (2021), using Wards distance with a 5% slanted band window in the R package dtwclust  (v5.5.10, Sarda-Espinosa 2022). A range of clustering solutions (number of clusters *k* = 2 – 10) were tested, with the ideal number of clusters determined by a majority vote from six internal cluster validity indices (Barbour et al. 2023). Dynamic time warp clustering may result in very fine-grained cluster solutions, as both the shape and mean values of a time series are evaluated (Hegg and Kennedy 2021). To account for clusters of fish distinguished solely based on differences in mean values between habitats that were otherwise ecologically similar (such as migratory fish from streams draining into lagoons of different average salinity, or brackish resident fish differing in mean δ^18^O residuals due to differences in mean temperature between lagoons), we grouped the resulting clusters further into a smaller set of ecologically informative groups, which we interpreted as behavioral phenotypes. To that end, a decision framework, grouping by capture location (e.g., pike captured in tributaries during spawning could not be brackish residents), natal origin (e.g., pike with Sr:Ca values corresponding to brackish water in the otolith core could not be freshwater resident), and lifelong habitat use (e.g., oscillations in Sr:Ca or δ^18^O residuals indicated habitat shifts), was applied (supplement, section C). To test whether these final behavioral phenotypes accurately represented natal (first year), early (second year) and later life (all remaining years) habitat use, we tested the reproducibility of behavioral phenotype assignments from average values of Sr:Ca and δ^18^O for the different life stages using jackknife cross-validation (MASS package, v7.3.57, Venables and Ripley 2002). The frequency distribution of behavioral phenotypes across capture locations was then assessed with a Χ^2^-test.

### Genetic population structure

To identify genotypes, we developed a genotyping assay based on a panel of 33 single nucleotide polymorphism (SNP) markers, targeting previously identified genomic candidate regions with maximal differentiation. To identify candidate genomic regions of maximal allele frequency difference that were likely involved in adaptive population divergence, we screened sequences of 11 DNA pools, representing samples of putative brackish, putative freshwater and putative anadromous populations by capture location (Roser et al. 2023), using whole-genome sequencing. Genes associated with these regions and their functions were identified based on an annotated pike genome (GCF_011004845.1, NCBI, 2020). Next, we individually genotyped all 1,514 pike, including 101 pike (*N* = 58 brackish, *N* = 43 tributary) for which both behavioral phenotype and genetic data were available. We used STRUCTURE (Pritchard et al. 2000) to determine the most likely number of genetic clusters and extract individual assignment probabilities to each genetic cluster. A PERMANOVA (vegan package, v2.6–2, Oksanen et al. 2022) tested the association between genotype and behavioral phenotypes, using assignment probabilities to the four genotypes as dependent variables. To assign discrete individual genotypes for frequency testing, an assignment probability threshold of 0.7 (Austrich et al. 2020; Skey et al. 2023) was applied, which offered a compromise between retaining individuals in the sample and applying a conservative threshold. We also tested the association of genotype, phenotype, and capture location with Χ^2^-tests. Behavioral phenotypes that differed significantly in their genotype assignment probability from all others, i.e., represented phenotypically and genetically distinct entities, were interpreted as ecotypes in the sense of Stronen et al. (2022).

### Growth analyses

To examine whether behavioral phenotypes and genotypes differed in age-specific growth and in response to thermosaline niche, we fitted linear mixed effect models to annual otolith increments. Behavioral phenotype, genotype, average annual δ^18^O residual as thermal proxy, average annual Sr:Ca as salinity proxy, age and sex were fixed effects, with a quadratic term for age, as growth slows down with age (von Bertalanffy 1938). Sex is a known predictor for growth in pike, with females growing larger than males (Casselman 1995). Individual ID was a random predictor, to account for the repeated measures design. To test for differences in age-specific growth rate, we included interactions between behavioral phenotype/genotype (run in separate models) and age. The model was run using restricted maximum likelihood estimation (lme4 & lmerTest packages, v. 1.1.30 and 3.1.3, Bates et al. 2015), and log-likelihood ratio (LLR) test for significance. Model assumptions were assessed graphically.

To infer lifelong growth performance, we estimated individual-level von Bertalanffy growth functions (von Bertalanffy 1938) separately for behavioral phenotypes, genotypes, and ecotypes, in a hierarchical Bayesian approach (Stan, version 2.21.0). Otolith radius *R* at age *t* was estimated

$$R_{(t,i)} = R_{\infty ,i} \left( {1 - {\varvec{e}}^{{ - {\varvec{k}}_{{{\mathbf{i}}}} ({\varvec{t}}_{{{\mathbf{i}}}} - {\varvec{t}}_{{0,{\mathbf{i}}}} }} } \right),$$

with *R*_t,i_ as the radius of fish *i* at age *t*. *R*_∞,i_ is the theoretical maximum radius, *k*_i_ is the Brody growth completion coefficient, *t*_*i*_ is the estimated age, and *t*_0,i_ is the age at which radius was zero for fish *i*. Radii at ages were nested within individuals, and individuals were nested within phenotypes/genotypes. Parameters of the models were assumed to be gamma-distributed with phenotype/genotype-specific mean and precision. Convergence problems and autocorrelation were assessed graphically (supplement, section F). Non-overlapping credibility intervals (95%) were interpreted as significant differences in lifetime growth among behavioral phenotypes, genotypes, or ecotypes.

## Results

### Behavioral phenotypes

**Fig. 2:**
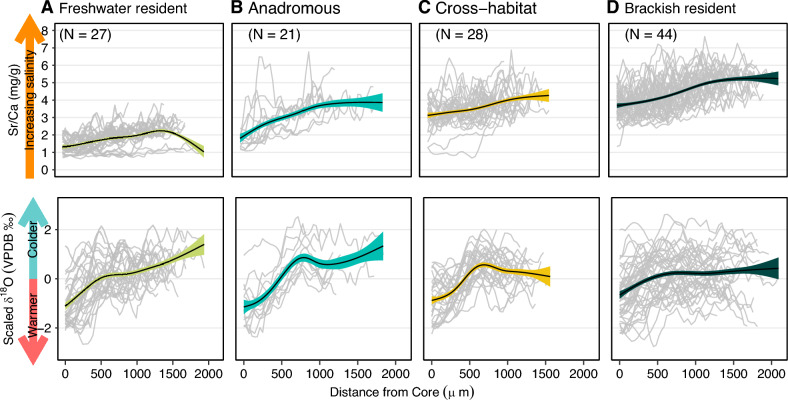
Behavioral phenotypes identified in northern pike (*Esox lucius*, *N* = 120), sampled between July 2019 and April 2022 from brackish lagoons and several freshwater tributaries around Rügen island, Germany. Upper panels show individual lifelong Sr/Ca values in grey in mg/g, fitted with a GAM smoothing Line. Lower panels show individual lifelong δ^18^O residuals in transparent grey fitted with a GAM smoothing line. The mean of the GAM smoother is shown as black line, colored areas depict 95% confidence intervals

Time-series clustering identified four clusters as the best solution for the lagoon sample and six clusters for the tributary sample. In the tributary sample, several clusters differed only in later-life Sr:Ca values, while in the lagoon sample, several clusters differed only in mean δ^18^O residuals, but not in the shape of their lifelong trajectories (Figs. S4, S5). To discern general habitat use patterns, clusters were further grouped based on natal origin, capture location and thermosaline history (supplement, section C). Through this approach, we identified four distinct behavioral phenotypes (Fig. 2):

(i) Freshwater residents (*N* = 27): Born in freshwater, these individuals exhibited low Sr/Ca values throughout life, suggesting freshwater residence, and a shift from warm (lower δ^18^O values) to colder (higher δ^18^O values) habitats with age (Fig. 2A).
(ii) Anadromous individuals (*N* = 21): Born in freshwater, these individuals oscillated between brackish and freshwater habitats in later life, with an ontogenetic shift from warm juvenile to colder adult habitats (Fig. 2B).
(iii) Brackish residents (*N* = 44): Born in brackish water, these individuals displayed high lifelong salinity, no freshwater signal and a weak ontogenetic shift from warm juvenile to colder adult habitats (Fig. 2D).
(iv) Cross-habitat phenotype (*N* = 28): Comprising pike captured from both freshwater tributaries and brackish lagoons, this phenotype had no clear freshwater or brackish origin. It instead exhibited intermediate lifelong salinity, a distinct ontogenetic shift from warm juvenile to colder adult habitats, and oscillations in salinity above the freshwater threshold (Fig. 2C).

**Fig. 3:**
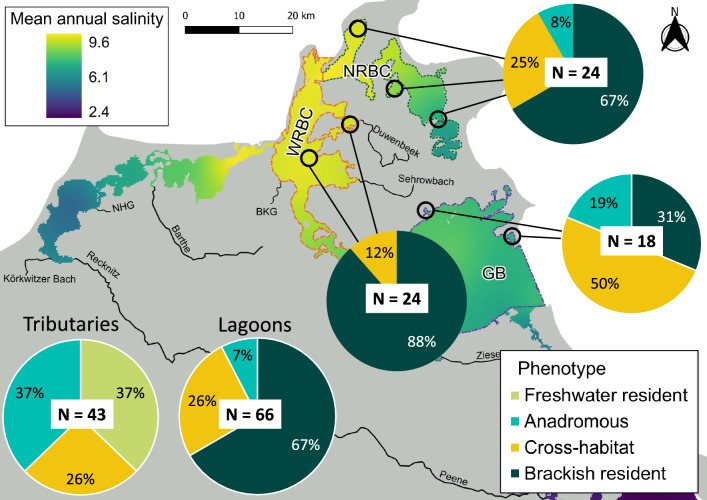
Distribution of behavioral phenotypes of northern pike (*Esox lucius*, *N* = 120), sampled between July 2019 and April 2022 in brackish lagoons and freshwater tributaries around Rügen island, Germany. *NRBC* North Rügen Bodden chain, *WRBC* West Rügen Bodden chain, *GB* Greifswalder Bodden, *NHG* Neuendorfer Hechtgraben, *BKG* Badendycksgraben. The two lower pie charts on the left represent the pooled samples for freshwater tributaries and brackish lagoons

These four behavioral phenotypes accurately reflected habitat use across life stages, with a high reproducibility rate based on life stage-specific Sr:Ca and δ^18^O residual values (82% correct jackknife reclassification). The ratio of males to females was constant across the behavioral phenotypes. In the lagoon sample (by capture location), 44 individuals (67%) were identified as brackish residents, 17 (26%) as cross-habitat, and 5 (7%) as anadromous. For the tributary sample, the timing of sampling was important. All individuals captured in tributaries outside spawning season (July 2019) were classified as freshwater residents (*N* = 11, Table S2), while those sampled during spawning season (*N* = 43) included relevant proportions of anadromous (N = 16, 37%) and cross-habitat types (*N* = 11, 26%), in addition to freshwater residents (*N* = 16, 37%, Fig. 3). The 11 individuals captured in tributaries outside of spawning season were omitted from frequency distribution tests. Frequency distributions of behavioral phenotypes of the remaining fish (*N* = 109) followed the salinity gradient (χ^2^ = 9.54, df = 2, *p* = 0.008): Fish captured from higher salinity lagoons were more likely brackish residents, while frequencies of cross-habitat and anadromous pike significantly increased from higher salinity to lower salinity lagoons and freshwater tributaries (Fig. 3).

### Genotypes

**Fig. 4:**
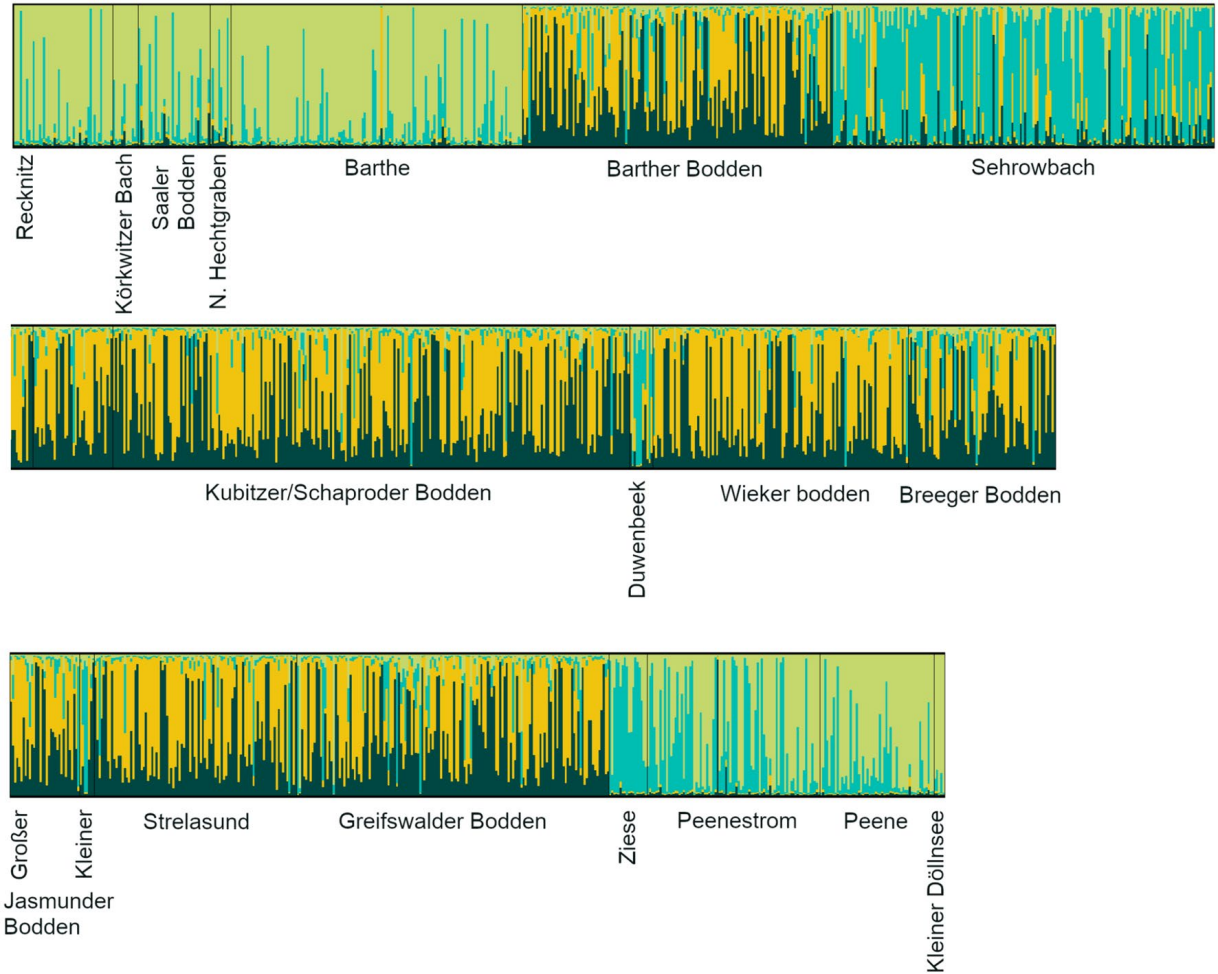
STRUCTURE plot of individual northern pike (*Esox lucius*, *N* = 1514), sampled between July 2019 and April 2022 in brackish lagoons and freshwater tributaries around Rügen island, Germany. Each vertical segmented line represents an individual pike. Sampling areas are ordered according to geographic location from west to east, and correspond to the sampling areas of Roser et al. (2023), described in detail in supplement, section D. The plot shows the best-fitting solution of *k* = 4 genetic clusters. Yellow and dark green corresponds to the putative brackish clusters brackish 1 and brackish 2, turquoise corresponds to the putative anadromous cluster, light green corresponds to the putative freshwater cluster

A genome-wide screen of 11 pooled sequence samples of pike revealed 33 candidate loci (supplement, section D, Table S4). Five SNP markers with high diagnostic potential between brackish water and freshwater samples were tightly associated with osmoregulatory genes, suggesting salinity contributed to the divergence (Table S4). Known functions of other candidate genes have not been associated with ecotypes in fishes yet (Table S4). STRUCTURE analysis revealed *k* = 4 clusters as the best solution (Fig. 4). We called these clusters  putative freshwater genotype, putative anadromous genotype and two putative brackish water genotypes brackish 1 and 2. Distribution of genotypes was correlated with capture location: The two putatively brackish genotypes (*N* = 13 for brackish 1; *N* = 22 for brackish 2) had mostly been captured in brackish lagoons (92%). Putatively freshwater genotypes (*N* = 19) had mostly been captured in the larger rivers Peene and Barthe (84%), and putatively anadromous genotypes (*N* = 17) had mostly been captured  in the smaller tributaries Sehrowbach and Ziese (76%) (χ^2^ = 81.84, df = 12, *p* < 0.0001, Fig. S12). 28 individuals did not reach the 0.7 assignment threshold and remained unassigned, suggesting they were related to more than one genotype.

### Age-specific and lifelong growth performance

The best performing age-specific growth model included *age* (continuous), *sex* (factor, 2 levels male/female), yearly mean δ^18^O residuals (*δ*^*18*^*O*_*res*_, continuous) as thermal marker, z-scored yearly mean Sr:Ca (Sr, continuous) as salinity marker, an interaction term between *age* and *behavioral phenotype* (factor, 4 levels) and the random effect of individual ID (*1|ID*, 120 groups). Genotype was not a significant predictor of growth (LLR = 12.45, *p* = 0.26, Fig. S13), and was therefore not included in the final model.

**Fig. 5:**
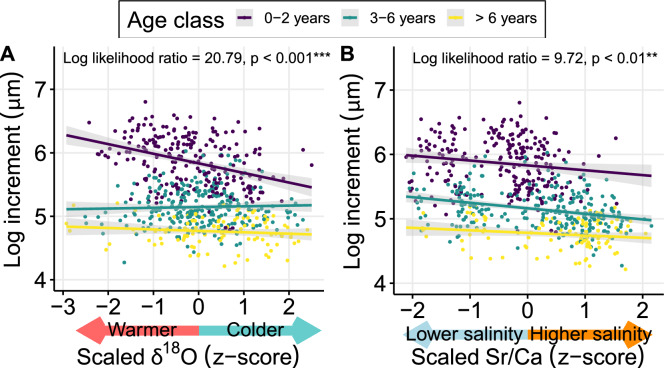
Effect of relative temperature (**A**, salinity-corrected δ^18^O values) and relative salinity (**B**, Sr/Ca values) on growth increments of northern pike (*Esox lucius*, *N* = 120), captured in brackish lagoons and freshwater tributaries around Rügen island between July 2019 and April 2022. Individuals were grouped into age categories: Early life (0–2 years), adult (3–6 years) and late adult (> 6 years), to visualize age- and stage-dependent effects. Colored lines represent the linear regression line between the predictor variable and the growth increments of each subgroup, and shaded areas around the regression lines depict the 95% confidence intervals. Note that no pairwise comparisons were run between discrete age classes in the model

**Fig. 6:**
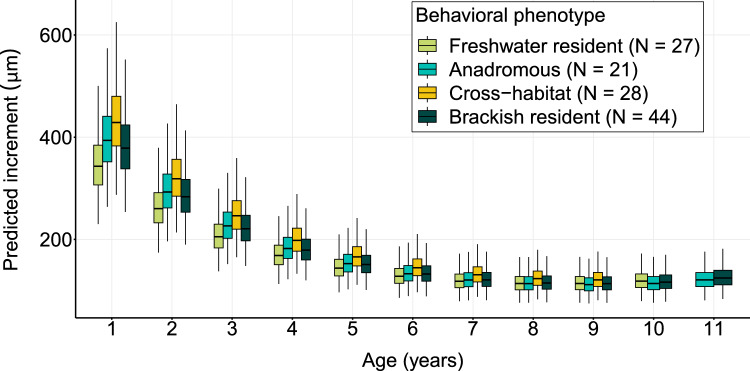
Predicted otolith increments for four behavioral phenotypes calculated from growth data of northern pike (*Esox lucius*, *N* = 120), sampled between July 2019 and April 2022 in the brackish lagoons and several freshwater tributaries around Rügen island in Germany. Boxes depict the median, lower, and upper quantile of the data, with vertical lines depicting the 95% confidence interval

**Fig. 7:**
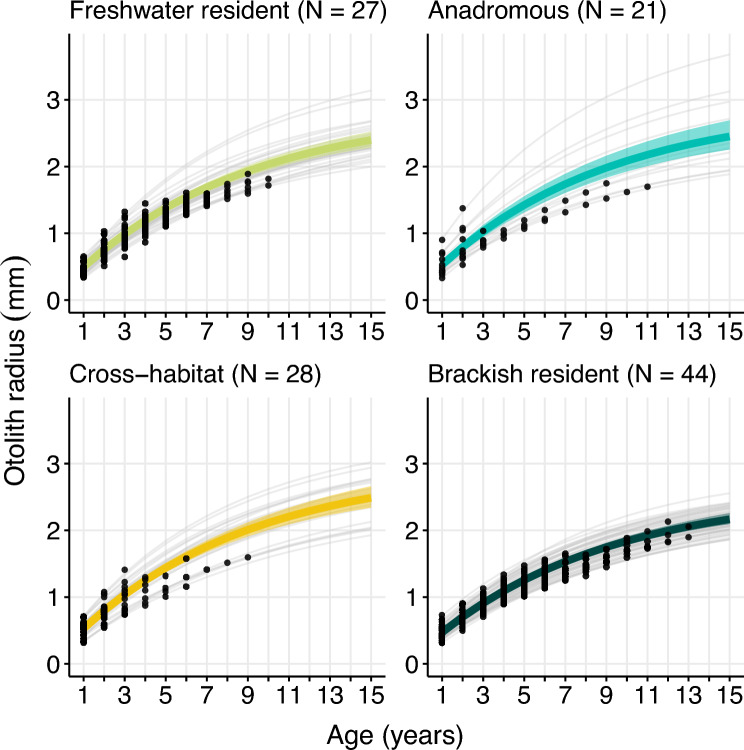
Von Bertalanffy growth curves of four behavioral phenotypes described in northern pike (*Esox lucius*, *N* = 120), sampled between July 2019 and April 2022 in brackish lagoons and freshwater tributaries around Rügen island, Germany. Grey lines represent individual-level growth curves. Colored lines represent mean radius-at-age of phenotypes, with shaded areas indicating 95% credible intervals

The model,

$$\log_{10} {\text{Increment}}\sim {\text{age}} + {\text{age}}^{2} + {\text{sex}} + \delta^{18} O_{res} + Sr + {\text{age}} \times {\text{phenotype}} + \left( {1|ID} \right),$$

explained 78.9% of the variance in otolith increments of the 120 pike individuals, 77.3% was explained by fixed predictors (Table 1). Age and sex were significant predictors: As expected, fish grew slower with age, and females grew faster than males (Table 1). Relative temperature (δ^18^O_res_) was a significant predictor, where warmer relative temperature led to faster growth (Table 1). This effect appeared to be mainly driven by the early growth phase and diminished as individuals grew older (Fig. 5). Salinities exceeding the population mean, assessed by Sr:Ca z-scores, had a negative effect on pike growth, consistent across the whole age range (Table 1; Fig. 5). Pike behavioral phenotypes showed different growth performance at different ages, as indicated by a significant interaction between phenotype and age. In early life, freshwater residents grew slower, and cross-habitat types grew faster compared to the other phenotypes (Table 1). However, growth differences levelled out in later life (Fig. 6). We found no differences in lifelong growth between behavioral phenotypes, as 95% credibility intervals overlapped between phenotype-specific von Bertalanffy parameter estimates for all phenotypes (Table 2; Fig. 7). Similarly, average lifelong growth showed no difference between genotypes, or ecotypes (Tables S5, S6; Figs. S14, S15).

**Table 1:** Effects of fixed and random predictors on a linear mixed effects model of log_10_-transformed otolith increment widths of northern pike (*N* = 120), sampled from brackish lagoons and freshwater tributaries around Rügen island in Germany between July 2019 and April 2022

log_10_-transformed Increment width (marginal R^2^ = 0.77; conditional R^2^ = 0.79)^1^				
Predictors	Estimate (± SE)	*t*-value	LLR	*p*-value
Intercept	2.76 (0.03)	85.67		
Age	– 0.15 (0.01)	– 15.13		
**Age**^**2**^	**0.01 (0.00)**	**12.90**	**142.08**	< 0.001 ***
**Mean d18O residuals**	**– 0.03 (0.01)**	**– 4.74**	**20.79**	< 0.001 ***
**Mean Sr/Ca (z-score)**	**– 0.04 (0.01)**	**– 3.19**	**9.72**	< 0.01 **
Phenotype [BW resident]	**– **0.02 (0.03)	**– **0.68		
Phenotype [FW resident]	**– **0.08 (0.03)	**– **2.71		
Phenotype [Cross-habitat]	0.04 (0.03)	1.07		
**Sex [male]**	**– 0.03 (0.01)**	**– 2.62**	**6.84**	< 0.01 **
Lifeyear * phenotype [BW resident]	**0.01 (0.01)**	**0.63**	**388.13**	< 0.001 ***
Lifeyear * phenotype [FW resident]	**0.01 (0.01)**	**1.52**	**388.13**	< 0.001 ***
Lifeyear * phenotype [Cross-habitat]	**0.01 (0.01)**	**0.11**	**388.13**	< 0.001 ***

Random Effects	Variance (± SD)	t-value	LLR	p-value
**ID**	**0.001 (0.03)**		**4.20**	**0.04**
Residual	0.012 (0.11)			

*SE* Standard error, *SD* Standard deviation, *LLR* Log-likelihood ratio. Significant effects are shown in bold

^1^Marginal *R*^2^ describes the proportion of the total variance explained by fixed effects in the model; conditional *R*^2^ describes the proportion of total variance explained by fixed and random effects combined in the model

**Table 2:** Phenotype-specific von Bertalanffy growth parameters of northern pike (N = 120), sampled between July 2019 and April 2022 from brackish lagoons and freshwater tributaries around Rügen island

Phenotype	L_∞_	k	t_0_
Freshwater resident	2.61 – 3.18 (2.85)	0.10 – 0.13 (0.12)	– 0.77 to – 0.46 (– 0.62)
Anadromous	2.60 – 3.09 (2.85)	0.11 – 0.14 (0.12)	– 0.77 to – 0.48 (– 0.63)
Cross-habitat	2.61 – 3.31 (2.96)	0.10 – 0.14 (0.12)	– 0.76 to – 0.52 (– 0.64)
Brackish resident	2.52 – 2.87 (2.68)	0.10 – 0.12 (0.11)	– 0.93 to – 0.69 (– 0.81)

Parameter values are given in the interquartile range from 2.5% to 97.5% credible parameter space. Values in brackets denote the median parameter estimate

### Matching behavioral phenotypes with genotypes to infer ecotypes

**Fig. 8:**
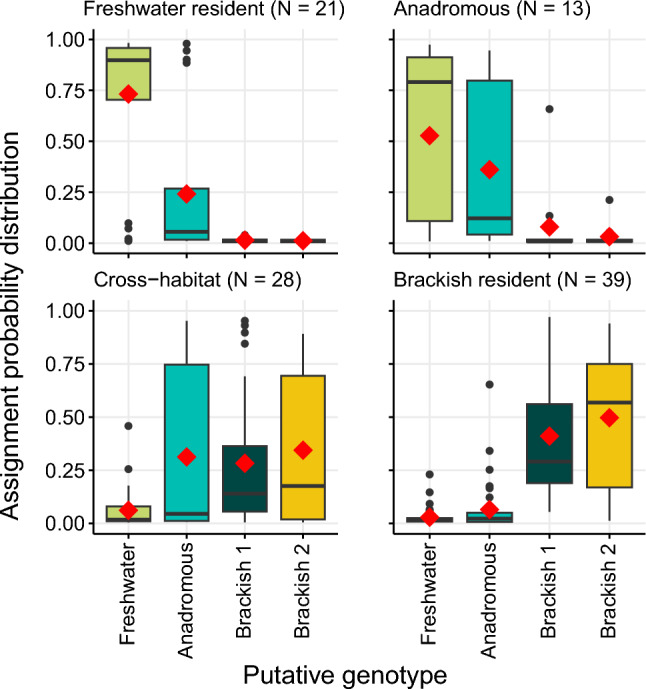
Assignment probabilities to four genetic clusters identified by STRUCTURE for four behavioral phenotypes described in northern pike (*Esox lucius*, *N* = 101), sampled from brackish lagoons and freshwater tributaries around Rügen island between July 2019 and April 2022. Boxes represent upper and lower quantile along with median assignment probability value per genotype, vertical lines represent the 95% confidence intervals, single points represent outlier values, and red diamonds indicate mean assignment probability per genotype

Behavioral phenotypes exhibited significantly different assignment probability distributions to the four genotypes (PERMANOVA with 9999 permutations, *F* = 19.55, *p* < 0.001), with behavioral phenotype explaining 37.8% of the variance in genotype assignment probabilities. Freshwater residents (*N* = 21) and anadromous phenotypes (*N* = 11) were related to both putative anadromous and freshwater genotypes, with no significant differences in assignment probabilities (pairwise PERMANOVA, fdr-adjusted pairwise Wilks λ tests, *p* = 0.20), but not to the putative brackish water genotypes (pairwise PERMANOVA, p_brackish_ = 0.0015, Fig. 8; S11). In contrast, brackish resident phenotypes (N = 39) were genetically distinct from all other phenotypes and predominantly comprised of the putative brackish genotypes 1 and 2 (pairwise PERMANOVA, p_anadromous_ = 0.0015; p_freshwater_ = 0.0015; p_cross-habitat_ = 0.0096) (Fig. 8; Fig. S11). Cross-habitat phenotypes (N = 28) comprised a mixture of putative anadromous and both brackish genotypes, with little relation to the putative freshwater genotype (Fig. 8; Fig. S11). The genotypic composition of the cross-habitat phenotype was significantly different from all others (pairwise PERMANOVA, p_anadromous_ = 0.0015; p_brackish_ = 0.0096; p_freshwater_ = 0.0015). No obvious patterns in behavioral phenotype expression (Fig. 8; Fig. S11), or capture location (Fig. S12), were evident for the two divergent putative brackish genotypes. Therefore, phenotype-genotype matching suggested the presence of three ecotypes: (i) a brackish ecotype encompassing two genotypes with limited gene flow and life-time residence in brackish areas, (ii) a freshwater ecotype expressing either freshwater residency or anadromy, and (iii) an intermediary cross-habitat ecotype adapted to intermediate salinity and limited reliance on freshwater. Lifelong growth of the three ecotypes was not significantly different (Fig. S15).

## Discussion

We integrated otolith microchemistry data on habitat use and migration behavior with genetic differentiation in a freshwater-adapted predatory fish, to test whether environmental gradients in salinity and temperature promote ecotype diversification in a brackish lagoon ecosystem. Our findings supported our first hypothesis, revealing significant ontogenetic variation in thermosaline niches among pike. They showed a preference for warmer and less saline habitats in early life, transitioning to colder, more saline environments as adults. In response to our second hypothesis, we identified four behavioral phenotypes: Freshwater residents, anadromous individuals, brackish residents, and a previously unrecognized cross-habitat phenotype. While phenotype-genotype matching confirmed the evolution of three ecotypes, not all phenotypes exhibited clear genetic differentiation. Freshwater and anadromous phenotypes were genetically similar, and all genotypes expressed more than one behavioral phenotype. Our results imply a split between a freshwater/anadromous and a brackish-adapted ecotype, with a third, intermediary, cross-habitat ecotype connecting them. Supporting our third hypothesis, we found evidence of clear separation in behavioral phenotypes and genotypes along the salinity gradient of the brackish lagoons. Divergent functional candidate genes related to osmoregulation suggested the observed differentiation was, at least in part, driven by adaptation to salinity.

Interindividual variability in habitat use revealed diverse migration and habitat use behaviors in the coastal pike meta-population. While previous studies have described three behavioral phenotypes in coastal pike—brackish residents, anadromous, and freshwater residents (e.g., Birnie‐Gauvin et al. 2019; Jacobsen et al. 2017; Möller et al. 2019; Nordahl et al. 2019)—we identified a fourth behavioral phenotype. This cross-habitat phenotype resembled intermediary behaviors connecting freshwater and marine realms reported in other coastal fishes (Almeida et al. 2023; Kerr et al. 2007, 2009; Limburg et al. 2001; Rohtla et al. 2020, 2023; Russell et al. 2022). Each behavioral phenotype likely faces specific trade-offs. Brackish residents avoid energetically costly spawning migrations and benefit from access to marine prey in the brackish lagoons (Winkler 1987). But they face risks of reproductive failure during high saline inflows (Sunde et al. 2018; Arlinghaus et al. 2023), and predation by top predators such as grey seals (*Halichoeres gryphus*) (Bergström et al. 2022; Olin et al. 2024). Freshwater residents also do not migrate and avoid the need for osmoregulatory adaptations. However, they may experience lower prey availability (Rohtla et al. 2012) and local environmental challenges, such as varying water levels and anoxic conditions in highly modified tributaries (Roser et al. 2023), reducing early growth. Anadromous individuals spawn in freshwater and benefit from productive brackish environments after outmigration (Rohtla et al. 2012). In turn, they face energy costs and increased mortality risk during migrations (Haugen et al. 2006). Cross-habitat individuals select intermediary habitats for spawning, such as sheltered bays with freshwater influence (Flink et al. 2023) and river mouths (Dhellemmes et al. 2023b; Lukyanova et al. 2024), potentially avoiding extensive migrations. Their offspring can then benefit from less saline conditions while retaining access to brackish environments, resulting in rapid early growth. However, the absence of old individuals in this phenotype suggests increased adult mortality, potentially offsetting early growth advantages (Roff 1988).

The positive effect of temperature on pike growth, particularly in early life, aligns with previous research (Pagel et al. 2015), suggesting that young pike thrive in sheltered habitats that warm up faster (Pursiainen et al. 2021). All phenotypes except freshwater residents experienced increasing salinities with age, which reflects higher osmoregulatory capacity in adults (Varsamos et al. 2005). This allows older and larger individuals to explore more saline habitats with a wider prey range (Winkler 1987). However, there is an upper limit to salinity adaptation (Jacobsen and Engström-Öst 2018), as higher salinities negatively affect growth, likely due to the energetic costs of osmoregulation in fluctuating salinities (Bœuf and Payan 2001). Our findings collectively suggest that pike undergo ontogenetic habitat shifts from less saline, warm habitats to open, more saline habitats, similar to habitat shifts from shallow to deeper habitats known from pike in lakes (Casselman and Lewis 1996), but also indicate that higher salinities can reduce growth despite evolutionary adaptations to brackish environments.

Our study builds upon prior research on coastal pike (e.g., Engstedt et al. 2010, 2014; Möller et al. 2019, 2020; Tibblin et al. 2015, 2016; Nordahl et al. 2019; Sunde et al. 2018, 2019, 2022) by linking behavioral phenotypes with underlying genotypes along a salinity gradient. Genetic differentiation among behavioral phenotypes suggests evolutionary adaptations to salinity, consistent with earlier findings (Arlinghaus et al. 2023; Jørgensen et al. 2010; Lukyanova et al. 2024; Sunde et al. 2018, 2022). In addition, IBD (Möller et al. 2020; Nordahl et al. 2019), adaptations to other ecological factors, such as temperature (Sunde et al. 2019), and habitat alterations (Eschbach et al. 2021; Roser et al. 2023), i.e., IBR, may all have contributed to the observed patterns. For instance, the blocking of freshwater tributaries since the late 1970s in the study region (Roser et al. 2023) likely increased selection pressure for the evolution of cross-habitat pike. Differences in phenotypic and genotypic frequencies were particularly evident at the extremes of the salinity gradient, indicating salinity adaptation, i.e., IBE, was a major driver of differentiation, consistent with previous studies (Sunde et al. 2022). Unexpectedly, we identified two divergent, spatially overlapping genotypes within the brackish lagoons. The absence of thermosaline niche differentiation among the two brackish genotypes suggests sympatric coexistence. Previous telemetry work in our study area hinted at subtle differences in thermal microhabitat between the two brackish genotypes, but these differences were not statistically significant (Nolte et al. 2023). Mechanisms for the reproductive isolation between the two brackish genotypes remain unclear and may involve other ecological factors not resolved by our work.

Our study did not provide conclusive evidence for lifelong growth advantages between the behavioral phenotypes, genotypes, and ecotypes. Similar growth rates can facilitate coexistence (Kobler et al. 2009), as growth strongly correlates with fitness in pike (Haugen et al. 2006). However, despite comparable growth rates, different phenotypes and genotypes might still vary in reproductive fitness due to different breeding success in fluctuating environments (Bell 2010). Controlled common garden experiments using offspring from wild-captured parents, either pure or hybrids, as well as large-scale tracking and offspring assignments to parents could provide insights into the environment-dependent reproductive fitness variation of the various phenotypes, genotypes and ecotypes.

The presence of two well-defined ecotypes in freshwater and brackish habitats, linked by a third intermediary cross-habitat ecotype, aligns with the theory of habitat selection and ecotype evolution in variable environments (Rosenzweig 1974; Brown 1990). The overlap in behavioral phenotype expression between putatively anadromous and freshwater genotypes suggests a single freshwater-adapted ecotype, expressing migratory or resident behavior, which may depend on both environmental (Olsson et al. 2006) and genetic cues (Vainikka et al. 2023), consistent with predictions from partial migration theory (Chapman et al. 2011). Indeed, telemetry work on putatively anadromous pike in our study area revealed flexibility in migration behavior among years, with some individuals migrating into tributaries in one year, but remaining in estuaries during spawning time in the next (Dhellemmes et al. 2023b). Genetic differentiation between freshwater and putatively anadromous pike likely arose from local adaptation, exacerbated by isolation by distance, natal homing and spawning site fidelity to specific streams (Engstedt et al. 2014; Nordahl et al. 2019). The well-defined brackish resident ecotype has adapted to complete its entire life cycle in brackish habitats, and is known to show the highest reproductive fitness at intermediate salinities (Arlinghaus et al. 2023). The third, less defined cross-habitat ecotype, is an intermediate between freshwater/anadromous and brackish residency. This ecotype might be a response to extensive blockage of freshwater tributaries in the 1970s (Roser et al. 2023). These habitat alterations likely caused selection pressures for intermediate behavioral strategies that seek out low salinity areas for spawning, consistent with previous studies showing rapid adaptive divergence in response to anthropogenic habitat alteration in pike (Bekkevold et al. 2015; Eschbach et al. 2021). Therefore, the cross-habitat ecotype potentially evolved as a hybrid between anadromous and brackish lagoon genotypes that thrives in intermediate salinities. A discrepancy in the proportion of brackish residents (68%) in our work compared to previous studies in the same region (98.7%, Möller et al. 2020) can be explained by our identification of the cross-habitat ecotype.

Our results challenge the dichotomous categorization of pike into just two ecotypes along the Baltic coast, suggesting a range of individual habitat use and migration behaviors connect the two behavioral endpoints of freshwater and brackish residency. Behavioral extremes correspond with the extremes of the salinity gradient, consistent with partial migration theory (Cagnacci et al. 2011; Chapman et al. 2011). Adding to similar observations in other coastal fish species (Almeida et al. 2023; Kerr et al. 2007, 2009; Limburg et al. 2001; Rohtla et al. 2020, 2023), we suggest intermediary behaviors and partial migration patterns are a common and often overlooked occurrence in coastal fish populations. Conservation of the pike population requires protecting the whole suit of intrapopulation diversity, to retain portfolio effects of population productivity in the face of environmental change (Schindler et al. 2010). In light of stressors associated with climate change, eutrophication, flow disruption, and migration barriers in the region (Roser et al. 2023), anadromous phenotypes, already rare in the study region (Möller et al. 2019, 2020; Roser et al. 2023), might eventually go extinct, decreasing phenotypic diversity and resilience of coastal pike populations (Schindler et al. 2010).

### Limitations

Our study system showed significant seasonal water δ^18^O fluctuations due to evaporation during our study period (Aichner et al. 2022). However, these were consistent across the area and unlikely to impact our relative thermal proxy. Our sampling design for the otolith microchemistry did not cover the oligohaline lagoons and only two streams were sampled at depth, potentially underrepresenting freshwater phenotypes. But we found strong genetic similarities between oligohaline lagoons and freshwater tributaries, suggesting our sample likely captured the phenotypic diversity present in the system, despite this limitation. Further, anadromous fish might be in rivers only for restricted periods of times (days or weeks, Dhellemmes et al. 2023b), which might not be sufficient to be detected in otoliths. However, the temporal resolution of otolith transects, particularly in the early years, reached up to 40 combined determinations per annulus, which we deemed sufficient for detecting freshwater excursions even on weekly scales. In addition, the semi-random sampling design of our study prevented us from arriving at unbiased estimates of phenotypic composition at the different capture locations. Nonetheless, the result of phenotype frequency in response to the salinity gradient should be robust. Another limitation was our clustering approach, which might have obscured subtle patterns in juvenile and adult habitat use. However, high jackknife reproducibility (82%) of behavioral phenotypes indicated an accurate representation of habitat use across ontogenetic stages. Finally, a limited within-group sample size may have biased results on age-specific and lifelong growth, so that smaller differences remain undetected. Indeed, other research in the region suggests that the lifetime growth of freshwater residents may be lower than that of brackish residents (Rittweg et al. 2023), but we only detected this effect in the juvenile life stage.

## Conclusions and implications

Our study suggests that a salinity gradient in lagoon ecosystems fostered intraspecific diversification of ecotypes with distinct realized thermosaline niches that show similar growth, indicating comparable fitness potential. Flexible migration and habitat use behavior, both across phenotypes but also ontogenetically, constitute an adaptation to variable local ecological factors and contribute to ecotype evolution. The notion of pike as stenohaline freshwater species that can be categorized into only two ecotypes in coastal habitats (anadromous vs. brackish resident) is challenged by our findings, suggesting the species can evolve intermediary migration and habitat use strategies, and complete its life cycle across a wider range of salinities. That said, the negative impact of above-average salinities on growth, as well as laboratory findings of reduced reproductive success at salinities exceeding 10 PSU in brackish-adapted pike (Arlinghaus et al. 2023), indicates an upper threshold for salinity tolerance in this species. From a conservation perspective, our findings highlight the importance of maintaining and, if possible, increasing access to freshwater tributaries through habitat restoration (Roser et al. 2023). This could maintain phenotypic and genotypic diversity and increase the resilience of the pike meta-population through portfolio effects (Schindler et al., 2010). Improving connectivity between brackish lagoons and freshwater tributaries can help sustain and increase the currently rare anadromous fish and would likely also be of use for the conservation and improvement of cross-habitat pike.

## Supplementary Information

Below is the link to the electronic supplementary material.

Supplementary file1 (DOCX 2199 KB)

## Data Availability

Data used for the analysis are available from 10.18728/igb-fred-908.0, R and Stan code used are available from https://github.com/Traveller-2909/Ecotype_analysis/.

## References

[CR1] Aichner B, Rittweg T, Schumann R, Dahlke S, Duggen S, Dubbert D (2022) Spatial and temporal dynamics of water isotopes in the riverine-marine mixing zone along the German Baltic Sea coast. Hydrol Process 36:e14686. 10.1002/hyp.14686

[CR2] Almeida R, Tanner SE, Mateus CS, Ribeiro F, Quintella BR (2023) Not so much a sea bass: Divergent European sea bass (*Dicentrarchus labrax* L.) freshwater incursions. J Fish Biol 104:1241–1246. 10.1111/jfb.15641 38148526 10.1111/jfb.15641

[CR3] Arlinghaus R, Rittweg T, Dhellemmes F, Koemle D, Gemert R, Schubert H, Niessner D, Möller S, Droll JS, Friedland R, Lewin WC, Dorow M, Westphal L, Ehrlich E, Strehlow HV, Weltersbach MS, Roser P, Braun M, Feldhege F, Winkler H (2023) A synthesis of a coastal northern pike (*Esox lucius*) fishery and its social-ecological environment: implications for management and research of pike in brackish lagoons in the southern Baltic Sea, Germany. Fisher Res 263:106663. 10.1016/j.fishres.2023.106663

[CR4] Austrich A, Mora MS, Mapelli FJ, Fameli A, Kittlein MJ (2020) Influences of landscape characteristics and historical barriers on the population genetic structure in the endangered sand-dune subterranean rodent *Ctenomys australis*. Genetica 148:149–164. 10.1007/s10709-020-00096-1 32451787 10.1007/s10709-020-00096-1

[CR5] Barbour N, Robillard AJ, Shillinger GL, Lyubchich V, Secor DH, Fagan WF, Bailey H (2023) Clustering and classification of vertical movement profiles for ecological inference of behavior. Ecosphere 14:e4384. 10.1002/ecs2.4384

[CR6] Bates D, Maechler M, Bolker B, Walker S (2015) Fitting linear mixed-effects models using lme4. J Stat Softw 67:1–48. 10.18637/jss.v067.i01

[CR7] Bekkevold D, Jacobsen L, Hemmer-Hansen J, Berg S, Skov C (2015) From regionally predictable to locally complex population structure in a freshwater top predator: River systems are not always the unit of connectivity in northern pike *Esox lucius*. Ecol Freshw Fish 24:305–316. 10.1111/eff.12149

[CR8] Bell G (2010) Fluctuating selection: The perpetual renewal of adaptation in variable environments. Philos Trans Royal Soc B Biol Sci 365:87–97. 10.1098/rstb.2009.0150 10.1098/rstb.2009.0150 PMC2842698 20008388

[CR9] Berggren H, Nordahl O, Tibblin P, Larsson P, Forsman A (2016) Testing for local adaptation to spawning habitat in sympatric subpopulations of pike by reciprocal translocation of embryos. PLoS ONE 11:e0154488. 10.1371/journal.pone.0154488 27139695 10.1371/journal.pone.0154488 PMC4854435

[CR10] Bergström U, Larsson S, Erlandsson M, Ovegård M, Ragnarsson Stabo H, Östman Ö, Sundblad G (2022) Long-term decline in northern pike (*Esox lucius* L.) populations in the Baltic Sea revealed by recreational angling data. Fisher Res 251:106307. 10.1016/j.fishres.2022.106307

[CR11] Birnie-Gauvin K, Birch HL, Kragh T, Jacobsen L, Aarestrup K (2019) Getting cosy in freshwater: Assumed to be brackish pike are not so brackish after all. Ecol Freshw Fish 28:376–384. 10.1111/eff.12460

[CR12] Blain SA, Schluter D, Adams CE, Amundsen P-A, Knudsen R, Chavarie L (2023) Patterns and repeatability of multi-ecotype assemblages of sympatric salmonids. Glob Ecol Biogeogr 32:2257–2270. 10.1111/geb.13763

[CR13] Bœuf G, Payan P (2001) How should salinity influence fish growth? Compar Biochem Physiol Part C 130:411–423. 10.1016/s1532-0456(01)00268-x 10.1016/s1532-0456(01)00268-x 11738629

[CR14] Brown JS (1990) Habitat selection as an evolutionary game. Evolution 44:732–746. 10.1111/j.1558-5646.1990.tb05951.x 28567976 10.1111/j.1558-5646.1990.tb05951.x

[CR15] Cagnacci F, Focardi S, Heurich M, Stache A, Hewison AJM, Morellet N, Kjellander P, Linell JDC, Mysterud A, Neteler M, Delucchi L, Ossi F, Urbano F (2011) Partial migration in roe deer: Migratory and resident tactics are end points of a behavioural gradient determined by ecological factors. Oikos 120:1790–1802. 10.1111/j.1600-0706.2011.19441.x

[CR16] Casselman J (1995) Age, growth and environmental requirements of pike. In: Craig (1995) Pike (1st Ed). Springer, Dordrecht. 69–101. 10.1007/978-94-015-8775-4

[CR17] Casselman JM, Lewis CA (1996) Habitat requirements of northern pike (*Esox lucius*). Can J Fish Aquat Sci 53:161–174. 10.1139/f96-019

[CR18] Chapman BB, Brönmark C, Nilsson JA, Hansson LA (2011) The ecology and evolution of partial migration. Oikos 120:1764–1775. 10.1111/j.1600-0706.2011.20131.x

[CR19] Clemens BJ, Schreck CB (2021) An assessment of terminology for intraspecific diversity in fishes, with a focus on “ecotypes” and “life histories”. Ecol Evol 11:10772–10793. 10.1002/ece3.7884 34429881 10.1002/ece3.7884 PMC8366897

[CR20] Darnaude AM, Sturrock A, Trueman CN, Mouillot D, Campana SE, Hunter E (2014) Listening in on the past: What can otolith δ^18^O values really tell us about the environmental history of fishes? PLoS ONE 9:e108539. 10.1371/journal.pone.0108539 25279667 10.1371/journal.pone.0108539 PMC4184789

[CR21] Delgado ML, Ruzzante DE (2020) Investigating diadromy in fishes and its loss in an -omics era. iScience 23:101837. 10.1016/j.isci.2020.101837 33305191 10.1016/j.isci.2020.101837 PMC7718486

[CR22] Dennenmoser S, Vamosi SM, Nolte AW, Rogers SM (2017) Adaptive genomic divergence under high gene flow between freshwater and brackish-water ecotypes of prickly sculpin (*Cottus asper*) revealed by Pool-Seq. Mol Ecol 26:25–42. 10.1111/mec.13805 27541083 10.1111/mec.13805

[CR23] Dhellemmes F, Aspillaga E, Rittweg T, Alós J, Möller P, Arlinghaus R (2023a) Body size scaling of space use in coastal pike (*Esox lucius*) in brackish lagoons of the southern Baltic Sea. Fish Res 260:106560. 10.1016/j.fishres.2022.106560

[CR24] Dhellemmes F, Lukyanova O, Roser P, Arlinghaus R (2023b) Anadromie und Homing von Boddenhechten. Berichte des IGB 33:277–296

[CR25] Diaz-Suarez A, Noreikiene K, Kisand V, Burimski O, Svirgsden R, Rohtla M, Ozerov M, Gross R, Vetemaa M, Vasemägi A (2022) Temporally stable small-scale genetic structure of northern pike (*Esox lucius*) in the coastal Baltic Sea. Fish Res 254:106402. 10.1016/j.fishres.2022.106402

[CR26] Doebeli M, Dieckmann U (2003) Speciation along environmental gradients. Nature 421:259–264. 10.1038/nature01274 12529641 10.1038/nature01274

[CR27] Durif CMF, Arts M, Bertolini F, Cresci A, Daverat F, Karlsbakk E, Koprivnikar J, Moland E, Olsen EM, Parzanini C, Power M, Rohtla M, Skiftesvik AB, Thorstad E, Vøllestad LA, Browman HI (2023) The evolving story of catadromy in the European eel (*Anguilla anguilla*). ICES J Mar Sci 80:2253–2265. 10.1093/icesjms/fsad149

[CR28] Engstedt O, Stenroth P, Larsson P, Ljunggren L, Elfman M (2010) Assessment of natal origin of pike (*Esox lucius*) in the Baltic Sea using Sr: Ca in otoliths. Environ Biol Fishes 89:547–555. 10.1007/s10641-010-9686-x

[CR29] Engstedt O, Engkvist R, Larsson P (2014) Elemental fingerprinting in otoliths reveals natal homing of anadromous Baltic Sea pike (*Esox lucius* L.). Ecol Freshw Fish 23:313–321. 10.1111/eff.12082

[CR30] Eschbach E, Nolte AW, Kohlmann K, Alós J, Schöning S, Arlinghaus R (2021) Genetic population structure of a top predatory fish (northern pike, *Esox lucius*) covaries with anthropogenic alteration of freshwater ecosystems. Freshw Biol 66:884–901. 10.1111/fwb.13684

[CR31] Felmy A, Reznick DN, Travis J, Potter T, Coulson T (2022) Life histories as mosaics: Plastic and genetic components differ among traits that underpin life-history strategies. Evolution 76:585–604. 10.1111/evo.14440 35084046 10.1111/evo.14440 PMC9303950

[CR32] Flink H, Tibblin P, Hall M, Hellström G, Nordahl O (2023) Variation among bays in spatiotemporal aggregation of Baltic Sea pike highlights management complexity. Fish Res 259:106579. 10.1016/j.fishres.2022.106579

[CR33] Forsman A, Tibblin P, Berggren H, Nordahl O, Koch-Schmidt P, Larsson P (2015) Pike *Esox lucius* as an emerging model organism for studies in ecology and evolutionary biology: A review. J Fish Biol 87:472–479. 10.1111/jfb.12712 26077107 10.1111/jfb.12712 PMC4744780

[CR34] Grimm MP (1981) The composition of northern pike (*Esox lucius* L.) populations in four shallow waters in the Netherlands, with special reference to factors influencing 0+ pike biomass. Aquac Res 12:61–76. 10.1111/j.1365-2109.1981.tb00011.x

[CR35] Haugen TO, Winfield IJ, Vøllestad LA, Fletcher JM, James JB, Stenseth NC (2006) The ideal free pike: 50 years of fitness-maximizing dispersal in Windermere. Proc R Soc B 273:2917–2924. 10.1098/rspb.2006.3659 17015363 10.1098/rspb.2006.3659 PMC1639511

[CR36] Hegg JC, Kennedy BP (2021) Let’s do the time warp again: Non-linear time series matching as a tool for sequentially structured data in ecology. Ecosphere 12:e03742. 10.1002/ecs2.3742

[CR37] Hendry AP, Day T (2005) Population structure attributable to reproductive time: Isolation by time and adaptation by time. Mol Ecol 14:901–916. 10.1111/j.1365-294x.2005.02480.x 15773924 10.1111/j.1365-294X.2005.02480.x

[CR38] Jacobsen L, Engström-Öst J (2018) Coping with environments; vegetation, turbidity and abiotics. In: Skov C, Nilsson PA (2018) Biology and ecology of pike (1st ed.). CRC Press, Boca Raton. 32–62. 10.1201/9781315119076

[CR39] Jacobsen L, Bekkevold D, Berg S, Jepsen N, Koed A, Aarestrup K, Baktoft H, Skov C (2017) Pike (*Esox lucius* L.) on the edge: Consistent individual movement patterns in transitional waters of the western Baltic. Hydrobiologia 784:143–154. 10.1007/s10750-016-2863-y

[CR40] Jørgensen AT, Hansen BW, Vismann B, Jacobsen L, Skov C, Berg S, Bekkevold D (2010) High salinity tolerance in eggs and fry of a brackish *Esox lucius* population. Fish Manage Ecol 17:554–560. 10.1111/j.1365-2400.2010.00755.x

[CR41] Kafemann R, Adlerstein S, Neukamm R (2000) Variation in otolith strontium and calcium ratios as an indicator of life-history strategies of freshwater fish species within a brackish water system. Fish Res 46:313–325. 10.1016/s0165-7836(00)00156-9

[CR42] Kerr LA, Secor DH, Kraus RT (2007) Stable isotope (δ^13^C and δ^18^O) and Sr/Ca composition of otoliths as proxies for environmental salinity experienced by an estuarine fish. Mar Ecol Prog Ser 349:245–253. 10.3354/meps07064

[CR43] Kerr LA, Secor DH, Piccoli PM (2009) Partial migration of fishes as exemplified by the estuarine-dependent white perch. Fisheries 34:114–123. 10.1577/1548-8446-34.3.114

[CR44] Kobler A, Klefoth T, Mehner T, Arlinghaus R (2009) Coexistence of behavioural types in an aquatic top predator: A response to resource limitation? Oecologia 161:837–847. 10.1007/s00442-009-1415-9 19609567 10.1007/s00442-009-1415-9

[CR45] Kültz D (2015) Physiological mechanisms used by fish to cope with salinity stress. J Exp Biol 218:1907–1914. 10.1242/jeb.118695 26085667 10.1242/jeb.118695

[CR46] Kusakabe M, Ishikawa A, Ravinet M, Yoshida K, Makino T, Toyoda A, Fujiyama A, Kitano J (2017) Genetic basis for variation in salinity tolerance between stickleback ecotypes. Mol Ecol 26:304–319. 10.1111/mec.13875 27706866 10.1111/mec.13875

[CR47] Laikre L, Miller LM, Palmé A, Palm S, Kapuscinski AR, Thoresson G, Ryman N (2005) Spatial genetic structure of northern pike (*Esox lucius*) in the Baltic Sea. Mol Ecol 14:1955–1964. 10.1111/j.1365-294x.2005.02570.x 15910318 10.1111/j.1365-294X.2005.02570.x

[CR48] Larsson P, Tibblin P, Koch-Schmidt P, Engstedt O, Nilsson J, Nordahl O, Forsman A (2015) Ecology, evolution, and management strategies of northern pike populations in the Baltic Sea. Ambio 44:451–461. 10.1007/s13280-015-0664-6 26022327 10.1007/s13280-015-0664-6 PMC4447694

[CR49] Limburg KE, Landergren P, Westin L, Elfman M, Kristiansson P (2001) Flexible modes of anadromy in Baltic sea trout: Making the most of marginal spawning streams. J Fish Biol 59:682–695. 10.1111/j.1095-8649.2001.tb02372.x

[CR50] Lindmark M, Ohlberger J, Gårdmark A (2022) Optimum growth temperature declines with body size within fish species. Glob Change Biol 28:2259–2271. 10.1111/gcb.16067 10.1111/gcb.16067 35060649

[CR51] Lukyanova O, Dhellemmes F, Dennenmoser S, Nolte AW, Arlinghaus R (2024) Combining biotelemetry and genetics provides complementary insights relevant to the management and conservation of a freshwater predator (*Esox lucius*) living in brackish lagoons. Aquat Sci 86:1–16. 10.1007/s00027-024-01090-x

[CR52] Maes GE, Van Houdt JKJ, De Charleroy D, Volckaert FAM (2003) Indications for a recent holarctic expansion of pike based on a preliminary study of mtDNA variation. J Fish Biol 63:254–259. 10.1046/j.1095-8649.2003.00140.x

[CR53] Magnuson JJ, Crowder LB, Medvick PA (1979) Temperature as an ecological resource. Am Zool 19:331–343. 10.1093/icb/19.1.331

[CR54] McRae BH (2006) Isolation by resistance. Evolution 60:1551–1561. 10.1111/j.0014-3820.2006.tb00500.x 17017056

[CR55] Miller LM, Kallemeyn L, Senanan W (2001) Spawning-site and natal-site fidelity by northern pike in a large lake: Mark–recapture and genetic evidence. Trans Am Fish Soc 130:307–316. 10.1577/1548-8659(2001)130%3c0307:Ssansf%3e2.0.Co;2

[CR56] Möller S, Winkler HM, Klügel A, Richter S (2019) Using otolith microchemical analysis to investigate the importance of brackish bays for pike (*Esox lucius* Linnaeus, 1758) reproduction in the southern Baltic Sea. Ecol Freshw Fish 28:602–610. 10.1111/eff.12478

[CR57] Möller S, Winkler HM, Richter S, Bastrop R (2020) Genetic population structure of pike (*Esox lucius* Linnaeus, 1758) in the brackish lagoons of the southern Baltic Sea. Ecol Freshw Fish 30:140–149. 10.1111/eff.12571

[CR58] Müller K (1986) Seasonal anadromous migration of the pike (*Esox lucius* L.) in coastal areas of the northern Bothnian Sea. Arch Hydrobiol 107:315–330. 10.1127/archiv-hydrobiol/107/1986/315

[CR59] Nolte WA, Dennenmoser S, Roser P, Aspillaga E, Rittweg T, Dhellemmes F, Möller S, Friedland R, Arlinghaus R (2023) Genetische Populationsstruktur. Berichte des IGB 33:312–342

[CR60] Nordahl O, Koch-Schmidt P, Sunde J, Yıldırım Y, Tibblin P, Forsman A, Larsson P (2019) Genetic differentiation between and within ecotypes of pike (*Esox lucius*) in the Baltic Sea. Aquat Conserv Mar Freshwat Ecosyst 29:1923–1935. 10.1002/aqc.3196

[CR61] Oksanen J, Simpson G, Blanchet F, Kindt R, Legendre P, Minchin P, O’Hara R, Solymos P, Stevens M, Szoecs E, Wagner H, Barbour M, Bedward M, Bolker B, Borcard D, Carvalho G, Chirico M, De Caceres M, Durand S, Evangelista H et al (2022) vegan: Community Ecology Package. R package version 2.6–2, https://CRAN.R-project.org/package=vegan.

[CR62] Olin AB, Bergström U, Bodin Ö, Sundblad G, Eriksson BK, Erlandsson M, Fredriksson R, Eklöf JS (2024) Predation and spatial connectivity interact to shape ecosystem resilience to an ongoing regime shift. Nat Commun 15:1304. 10.1038/s41467-024-45713-1 38347008 10.1038/s41467-024-45713-1 PMC10861472

[CR63] Olsson IC, Greenberg LA, Bergman E, Wysujack K (2006) Environmentally induced migration: The importance of food. Ecol Lett 9:645–651. 10.1111/j.1461-0248.2006.00909.x 16706909 10.1111/j.1461-0248.2006.00909.x

[CR64] Pagel T, Bekkevold D, Pohlmeier S, Wolter C, Arlinghaus R (2015) Thermal and maternal environments shape the value of early hatching in a natural population of a strongly cannibalistic freshwater fish. Oecologia 178:951–965. 10.1007/s00442-015-3301-y 25894093 10.1007/s00442-015-3301-y

[CR65] Palder J, Radinger J, Droll J, Arlinghaus R (2023) Laichzeiten, Reifungslängen und Fruchtbarkeit. Berichte des IGB 33:184–193

[CR66] Patterson WP, Smith GR, Lohmann KC (1993) Continental paleothermometry and seasonality using the isotopic composition of aragonitic otoliths of freshwater fishes. Geophys Monogr Ser 78:191–202. 10.1029/GM078p0191

[CR67] Pörtner HO, Schulte PM, Wood CM, Schiemer F (2010) Niche dimensions in fishes: An integrative view. Physiol Biochem Zool 83:808–826. 10.1086/655977 20704490 10.1086/655977

[CR68] Pritchard JK, Stephens M, Donnelly P (2000) Inference of population structure using multilocus genotype data. Genetics 155:945–959. 10.1093/genetics/155.2.945 10835412 10.1093/genetics/155.2.945 PMC1461096

[CR69] Pursiainen A, Veneranta L, Kuningas S, Saarinen A, Kallasvuo M (2021) The more sheltered, the better – coastal bays and lagoons are important reproduction habitats for pike in the northern Baltic Sea. Estuar Coast Shelf Sci 259:107477. 10.1016/j.ecss.2021.107477

[CR70] Rittweg T, Palder J, Braun M, Arlinghaus R, Möller S, Winkler H (2023) Wachstum, Kondition und Ernährung von Boddenhechten früher und heute. Berichte des IGB 33:193–232

[CR71] Rittweg TD, Trueman C, Ehrlich E, Wiedenbeck M, Arlinghaus R (2024) Corroborating otolith age using oxygen isotopes and comparing outcomes to scale age: Consequences for estimation of growth and reference points in northern pike (*Esox lucius*). Fish Manage Ecol 31:e12646. 10.1111/fme.12646

[CR72] Roff DA (1988) The evolution of migration and some life history parameters in marine fishes. Environ Biol Fishes 22:133–146. 10.1007/bf00001543

[CR73] Roff D (2002) Life history evolution (1st Ed). Sinauer Associates. 10.1016/B978-0-12-384719-5.00087-3

[CR74] Rohtla M, Vetemaa M, Urtson K, Soesoo A (2012) Early life migration patterns of Baltic Sea pike *Esox lucius*. J Fish Biol 80:886–893. 10.1111/j.1095-8649.2012.03226.x 22471807 10.1111/j.1095-8649.2012.03226.x

[CR75] Rohtla M, Matetski L, Taal I, Svirgsden R, Kesler M, Paiste P, Vetemaa M (2020) Quantifying an overlooked aspect of partial migration using otolith microchemistry. J Fish Biol 97:1582–1585. 10.1111/jfb.14522 32880933 10.1111/jfb.14522

[CR76] Rohtla M, Daverat F, Arts MT, Browman HI, Parzanini C, Skiftesvik AB, Thorstad EB, van der Meeren T, Vøllestad LA, Durif CMF (2023) Habitat use and growth of yellow-stage European eel in coastal and freshwater ecosystems in Norway. Can J Fish Aquat Sci 80:14–26. 10.1139/cjfas-2022-0033

[CR77] Rosenzweig ML (1974) On the evolution of habitat selection. In: Proceedings of the first international congress of ecology, TheHague 401–404. https://edepot.wur.nl/320441#page=395

[CR78] Roser P, Dhellemmes F, Rittweg T, Möller S, Winkler H, Lukyanova O, Niessner D, Schütt J, Kühn C, Dennenmoser S, Nolte AW, Radinger J, Koemle D, Arlinghaus R (2023) Synthesizing historic and current evidence for anadromy in a northern pike (*Esox lucius* L) meta-population inhabiting brackish lagoons of the southern Baltic Sea, with implications for management. Fish Res 260:106560. 10.1016/j.fishres.2023.106670

[CR79] Russell A, Taylor MD, Barnes TC, Johnson DD, Gillanders BM (2022) Habitat transitions by a large coastal sciaenid across life history stages, resolved using otolith chemistry. Mar Environ Res 176:105614. 10.1016/j.marenvres.2022.105614 35381507 10.1016/j.marenvres.2022.105614

[CR80] Sarda-Espinosa A (2022). dtwclust: Time Series Clustering Along with Optimizations for the Dynamic Time Warping Distance. R package version 5.5.10, https://CRAN.R-project.org/package=dtwclust.

[CR81] Schindler DE, Hilborn R, Chasco B, Boatright CP, Quinn TP, Rogers LA, Webster MS (2010) Population diversity and the portfolio effect in an exploited species. Nature 465:609–612. 10.1038/nature09060 20520713 10.1038/nature09060

[CR82] Skey ED, Ottewell KM, Spencer PB, Shaw RE (2023) Empirical landscape genetic comparison of single nucleotide polymorphisms and microsatellites in three arid-zone mammals with high dispersal capacity. Ecol Evol 13:e10037. 10.1002/ece3.10037 37153020 10.1002/ece3.10037 PMC10154367

[CR83] Sokolova I (2021) Bioenergetics in environmental adaptation and stress tolerance of aquatic ectotherms: Linking physiology and ecology in a multi-stressor landscape. J Experim Biol. 10.1242/jeb.236802 10.1242/jeb.236802 33627464

[CR84] Stronen AV, Norman AJ, Vander Wal E, Paquet PC (2022) The relevance of genetic structure in ecotype designation and conservation management. Evol Appl 15:185–202. 10.1111/eva.13339 35233242 10.1111/eva.13339 PMC8867706

[CR85] Sunde J, Tamario C, Tibblin P, Larsson P, Forsman A (2018) Variation in salinity tolerance between and within anadromous subpopulations of pike (*Esox lucius*). Sci Rep 8:1–22. 10.1038/s41598-017-18413-8 29311634 10.1038/s41598-017-18413-8 PMC5758576

[CR86] Sunde J, Larsson P, Forsman A (2019) Adaptations of early development to local spawning temperature in anadromous populations of pike (*Esox lucius*). BMC Evol Biol 19:1–13. 10.1186/s12862-019-1475-3 31331267 10.1186/s12862-019-1475-3 PMC6647320

[CR87] Sunde J, Yıldırım Y, Tibblin P, Bekkevold D, Skov C, Nordahl O, Larsson P, Forsman A (2022) Drivers of neutral and adaptive differentiation in pike (*Esox lucius*) populations from contrasting environments. Mol Ecol 31:1093–1110. 10.1111/mec.16315 34874594 10.1111/mec.16315

[CR88] Tibblin P, Forsman A, Koch-Schmidt P, Nordahl O, Johannessen P, Nilsson J, Larsson P (2015) Evolutionary divergence of adult body size and juvenile growth in sympatric subpopulations of a top predator in aquatic ecosystems. Am Nat 186:98–110. 10.1086/681597 26098342 10.1086/681597

[CR89] Tibblin P, Forsman A, Borger T, Larsson P (2016) Causes and consequences of repeatability, flexibility and individual fine-tuning of migratory timing in pike. J Anim Ecol 85:136–145. 10.1111/1365-2656.12439 26412457 10.1111/1365-2656.12439

[CR90] Trueman CN, MacKenzie KM, Palmer MR (2012) Identifying migrations in marine fishes through stable-isotope analysis. J Fish Biol 81:826–847. 10.1111/j.1095-8649.2012.03361.x 22803737 10.1111/j.1095-8649.2012.03361.x

[CR500] Vainikka A, Elvidge CK, Prokkola JM, Lemopoulos A, Vornanen M, Härkönen LS, Alioravainen N, Hyvärinen P (2023) Two-generation common-garden experiment reveals a strong genetic contribution to migration tendency in brown trout (Salmo trutta). Can J of Fish Aquat Sci 80:1394–1409. 10.1139/cjfas-2022-0304

[CR91] Varsamos S, Nebel C, Charmantier G (2005) Ontogeny of osmoregulation in postembryonic fish: A review. Compar Biochem Physiol Part A 141:401–429. 10.1016/j.cbpb.2005.01.013 10.1016/j.cbpb.2005.01.013 16140237

[CR92] Venables WN, Ripley BD (2002) Modern Applied Statistics with S (4th Ed.). Springer. 10.1007/978-0-387-21706-2

[CR93] von Bertalanffy L (1938) A quantitative theory of organic growth (inquiries on growth laws. II). Human Biology 10:181–213. https://www.jstor.org/stable/41447359

[CR94] Wang IJ, Bradburd GS (2014) Isolation by environment. Mol Ecol 23:5649–5662. 10.1111/mec.12938 25256562 10.1111/mec.12938

[CR95] Wąs-Barcz A, Bernaś R, Greszkiewicz M, Lejk AM, Fey DP (2023) Genetic structure of pike (*Esox lucius* Linnaeus, 1758) populations along the Polish coast of the southern Baltic Sea: Comparison to Danish brackish population. Fish Res 264:106709. 10.1016/j.fishres.2023.106709

[CR96] Wennerström L, Olsson J, Ryman N, Laikre L (2017) Temporally stable, weak genetic structuring in brackish water northern pike (*Esox lucius*) in the Baltic Sea indicates a contrasting divergence pattern relative to freshwater populations. Can J Fish Aquat Sci 74:562–571. 10.1139/cjfas-2016-0039

[CR97] Werner EE (1988) Size, scaling, and the evolution of complex life cycles. In: Ebenman B, Persson L (eds) Size-structured populations. Springer, Berlin, Heidelberg. 10.1007/978-3-642-74001-5_6

[CR98] Wilson KL, Matthias BG, Barbour AB, Ahrens RNM, Tuten T, Allen MS (2015) Combining samples from multiple gears helps to avoid fishy growth curves. North Am J Fish Manag 35:1121–1131. 10.1080/02755947.2015.1079573

[CR99] Winkler HM (1987) Einige Bemerkungen zur Ernährung des Hechtes (*Esox lucius* L.) in den Küstengewässern der DDR. Wissenschaftliche Zeitschrift der Wilhelm-Piek-Universität Rostock, Naturwissenschaften 36:53–56

[CR100] Wright S (1943) Isolation by distance. Genetics 28:114–138. 10.1093/genetics/28.2.114 17247074 10.1093/genetics/28.2.114 PMC1209196

