# Supplementary material for: Variable habitat use supports fine-scale population differentiation of a freshwater piscivore (northern pike, Esox lucius) along salinity gradients in brackish lagoons
Source: Oecologia. 2024 Oct 18;206(3-4):275–92. doi: 10.1007/s00442-024-05627-7 (PMC11599437; doi:10.1007/s00442-024-05627-7)
Supplement: Supplementary file 1 — Supplementary file1 (DOCX 2199 KB) [file 442_2024_5627_MOESM1_ESM.docx]

Variable habitat use supports fine-scale population differentiation of a freshwater piscivore (northern pike, *Esox lucius*) along salinity gradients in brackish lagoons

**Electronic supplementary material**

Timo D. Rittweg^a,g*^, Clive Trueman^b^, Michael Wiedenbeck^c^, Jan Fietzke^d^, Christian Wolter^a^, Lauren Talluto^e^, Stefan Dennenmoser^f^, Arne Nolte^f^, Robert Arlinghaus^a,g^

*^a^Leibniz Institute of Freshwater Ecology and Inland Fisheries (IGB), Müggelseedamm 310, 12587 Berlin, Berlin, Germany*

*^b^School of Ocean and Earth Science, University of Southampton Waterfront Campus, European Way, SO143ZH Southampton, UK*

*^c^German Research Center for Geosciences (GFZ) Potsdam, Telegrafenberg, 14473 Potsdam, Brandenburg, Germany*

*^d^GEOMAR Helmholtz Center for Ocean Research Kiel, Wischhofstr. 1-3, 24148 Kiel, Schleswig-Holstein, Germany*

*^e^Research Group Fluvial Ecosystem Ecology, Department of Ecology, University of Innsbruck, Technikerstr. 25, A-6020 Innsbruck, Austria*

*^f^Working group Ecological Genomics, Institute of Biology and Environmental Sciences, Carl von Ossietzky Universität Oldenburg, Carl von Ossietzky-Str. 9-11, 26111 Oldenburg, Germany*

*^g^Division of Integrative Fisheries Management, Faculty of Life Sciences, Humboldt-Universität zu Berlin, Unter den Linden 6, 10099 Berlin, Germany*

*Corresponding author: Timo D. Rittweg

E-Mail address: timo.rittweg@igb-berlin.de

Müggelseedamm 310, 12587 Berlin

## Section A: Study sites and sampling

To interpolate annual average salinity and temperature and average standard deviations across the study area, we averaged the environmental parameters salinity, temperature, from monthly measurements of the environmental monitoring carried out by the Landesamt für Umwelt, Naturschutz und Geologie in Mecklenburg-Vorpommern (LUNG MV, https://www.umweltkarten.mv-regierung.de/). We further calculated average values for total phosphorus and chlorophyll α content as a measure of productivity (**Table S1**), however, the data available for these parameters was limited to a few selected stations, so we did not use them for spatial interpolations. We used inverse path distance weighting (package ipdw, v1.0-0, Stachelek, 2022) to produce raster layers of interpolated salinity and temperature values for each month between 2005 and 2022 on a 250 m^2^ grid across the study area. We calculated mean and standard deviation rasters for the period between January 2005 (putative birth year of the oldest pike in our sample) and December 2022 (last year in which pike were sampled for this study) using the function rastercalc (package raster, v3.5-29, Hijmans, 2022)(**Fig. S1**).

**Table S1**. Summary of environmental and geographic parameters of the lagoons and three tributaries for which monitoring data was available, averaged over the studied area and time span covered by this study. Environmental parameters were averaged between January 2005 and December 2022. WRBC: Western Rügen Bodden Chain; NRBC: Northern Rügen Bodden Chain; GB: Greifswalder Bodden (data source: LUNG MV)

|  | **WRBC** | **NRBC** | **GB** | **Barthe** | **Sehrowbach** | **Ziese** |
| --- | --- | --- | --- | --- | --- | --- |
| **Area (km2)** | 231 | 159.4 | 540.1 | NA | NA | NA |
| **Mean depth (m)** | 1.8 | 3.5 | 5.8 | NA | NA | NA |
| **Max depth (m)** | 7.6 | 10.3 | 13.5 | NA | NA | NA |
| **Catchment area (km2)** | NA | 312 | 665 | 292 | NA | NA |
| **Water temperature (°C)** | 12.3±6.7 | 9.9±6.5 | 11.7±6.8 | 10.6±7.0 | 10.3±6.5 | 10.2±6.1 |
| **Salinity (PSU)** | 9.0±1.5 | 8.0±1.2 | 6.5±0.6 | 0.7±0.01 | 0.9±0.01 | 0.4±0.01 |
| **Secchi depth (m)** | 1.9±0.8 | 1.2±0.8 | 1.7±0.8 | NA | NA | NA |
| **Total phosphorus (µg/l)** | 40±19.6 | 64.2±39.1 | 45.8±21.1 | 223.7±194.6 | 169.4±132.6 | 126.5±86.6 |
| **Chlorophyll α (mg/m^3^)** | 7.9±6.9 | 34.6±37.6 | 14.6±13.6 | 51.9 ± 67.8 | 7.7±5.7 | 6.6±6.8 |


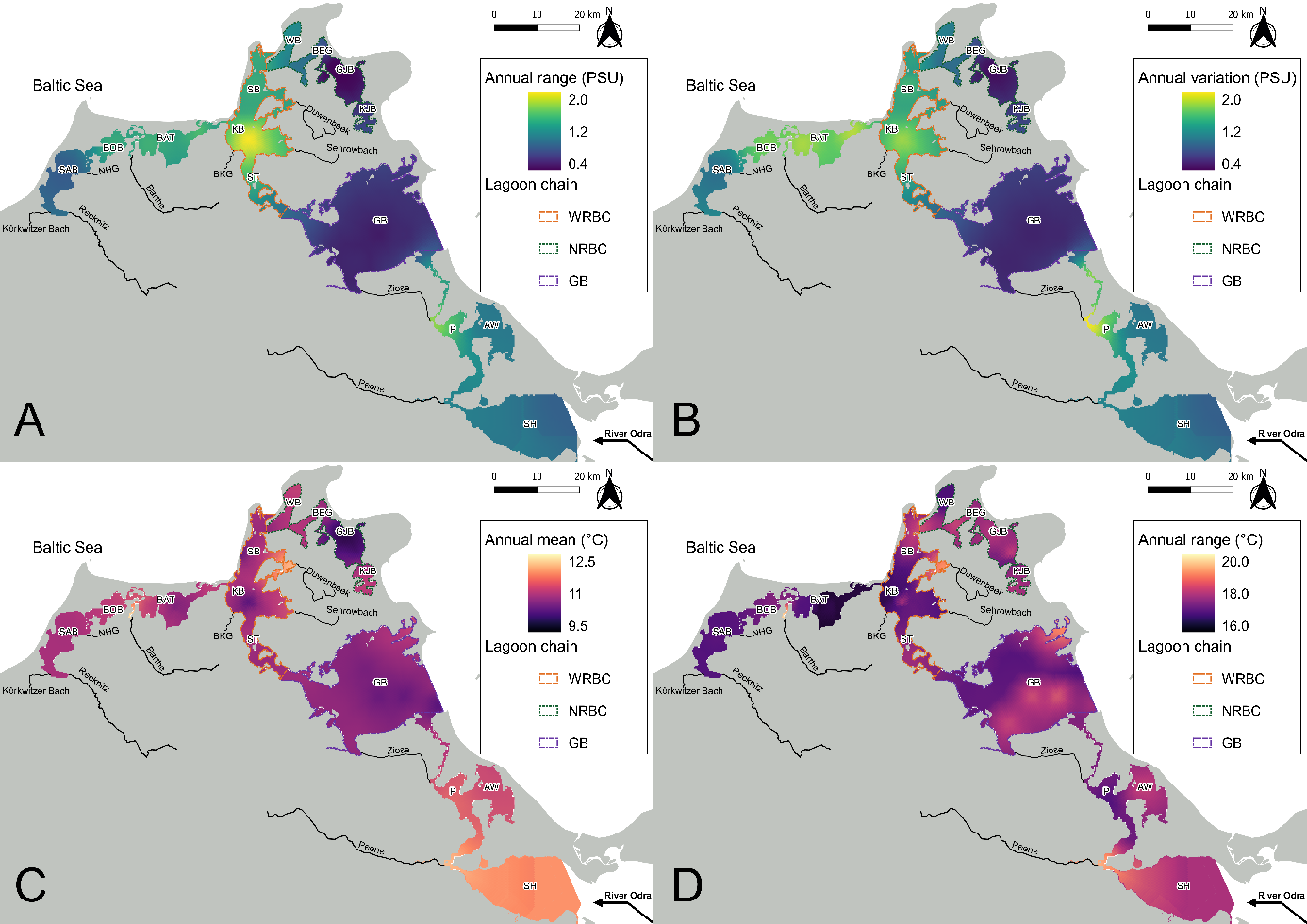


**Fig. S1** Salinity and temperature maps of the brackish lagoons around Rügen island, Germany, interpolated for the time between January 2005 and December 2022. A: Annual salinity range in PSU; B: Annual salinity variation (monthly standard deviation) in PSU; C: Annual mean temperature in °C; D: Annual temperature range in °C. Single lagoon abbreviations (from west to east): SAB: Saaler Bodden; BOB: Bodstedter Bodden; BAT: Barther Bodden & Grabow; KB: Kubitzer Bodden; SB: Schaproder Bodden; ST: Strelasund; WB: Wieker Bodden; BEG: Breeger Bodden; GJB: Großer Jasmunder Bodden; KJB: Kleiner Jasmunder Bodden; P: Peenestrom; AW: Achterwasser; SH: Stettiner Haff. Tributary abbreviations: NHG: Neuendorfer Hechtgraben; BKG: Badendycksgraben

**Table S2**. Overview of the sampling effort to sample the phenotypic diversity of northern pike from the brackish lagoons and freshwater tributaries around Rügen island in Germany between July 2019 and April 2022, indicating date and place of capture along with employed gear

| **Capture area** | **Sampling date** | **N** | **Gear** |
| --- | --- | --- | --- |
| WRBC | 01.11.2019 – 31.12.2019 | 12 | Fyke |
| WRBC | 01.01.2020 – 18.02.2020 | 10 | Gillnet |
| WRBC | 01.05.2020 – 31.05.2020 | 2 | Fyke |
| NRBC | 01.01.2020 – 28.02.2020 | 21 | Gillnet/Fyke |
| NRBC | 01.05.2020 – 31.05.2020 | 4 | Angling |
| GB | 01.11.2019 – 30.11.2019 | 2 | Angling |
| GB | 01.11.2020 – 31.01.2021 | 16 | Angling/Gillnet |
| Barthe | 01.07.2019 – 31.07.2019 | 6 | Electrofishing |
| Barthe | 01.03.2022 – 31.04.2022 | 15 | Electrofishing |
| Peene | 01.07.2019 – 31.07.2019 | 5 | Electrofishing |
| Sehrowbach | 01.04.2021 – 31.04.2021 | 5 | Electrofishing |
| Sehrowbach | 01.03.2022 – 31.04.2022 | 15 | Electrofishing |
| Ziese | 01.04.2021 – 31.04.2021 | 5 | Electrofishing |
| NHG | 01.04.2021 – 31.04.2021 | 3 | Electrofishing |
| Badendycksgraben | 01.03.2021 – 31.03.2021 | 2 | Electrofishing |


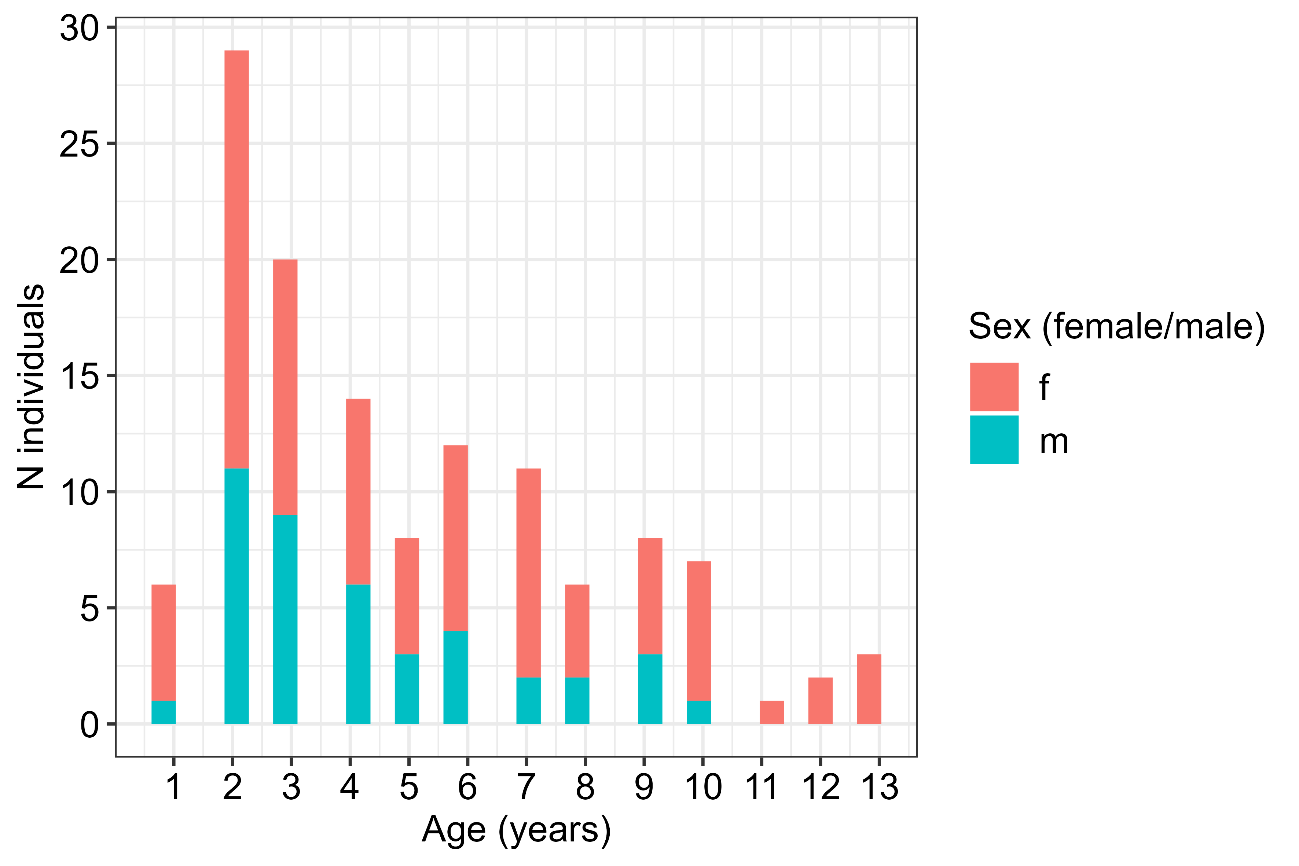


**Fig. S2** Age and sex distribution of northern pike (*Esox lucius*, N = 120) captured in brackish lagoons and freshwater tributaries around Rügen island in Northern Germany between July 2019 and April 2022


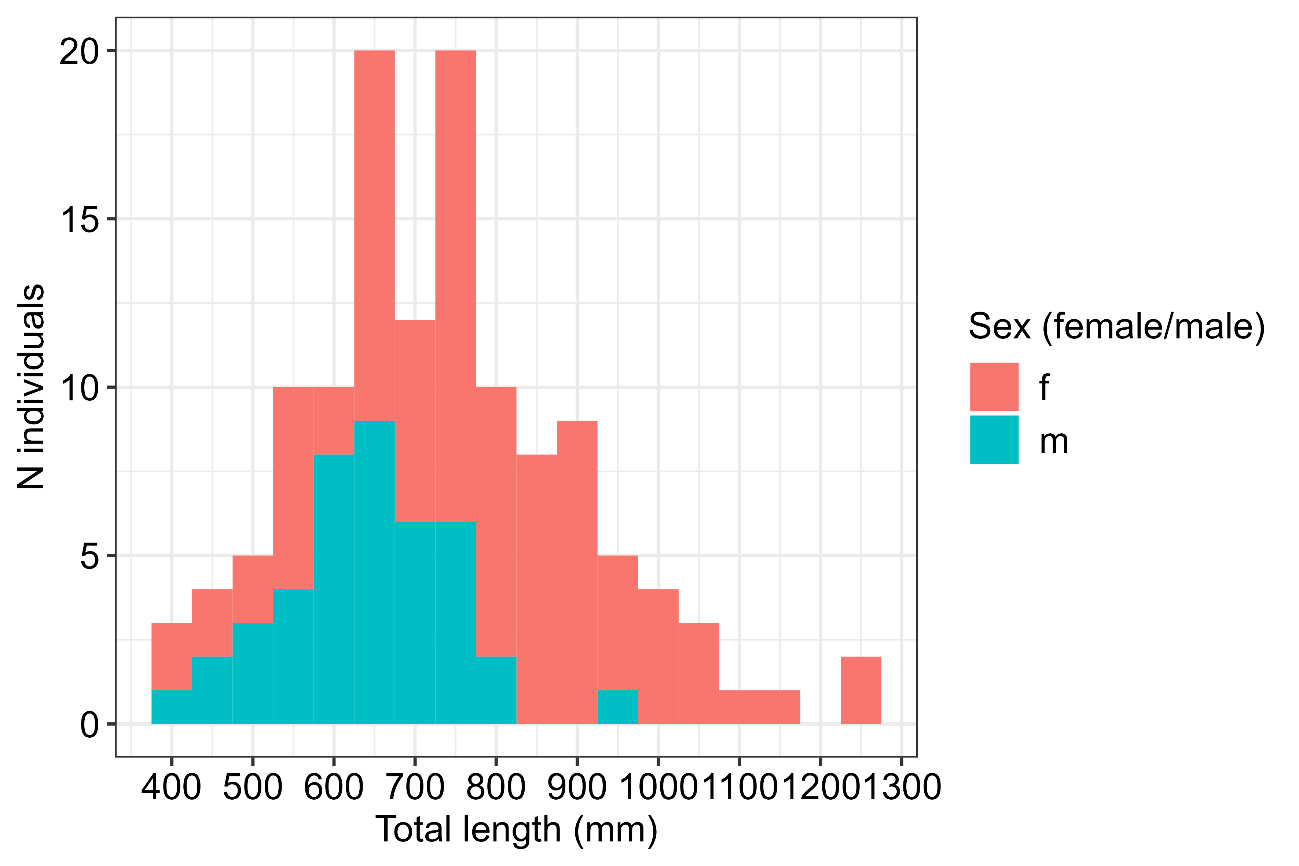


**Fig. S3** Size (total length, mm) and sex distribution of northern pike (*Esox lucius*, N = 120) captured in brackish lagoons and freshwater tributaries around Rügen island in Northern Germany between July 2019 and April 2022

## Section B: Otolith processing and microchemical analyses

Sagittal otoliths were extracted, cleaned, dried and glued to glass slides with Crystalbond glue. 100 µm thin sections were cut aiming for the otolith core using a Buehler Isomet Ltd 11-1180 low speed saw. The Crystalbond was dissolved with LC-MS grade Acetone (ROTISOLV®), producing free otolith thin sections. These sections were polished with 3000 and 5000 grit sandpaper, then sent to the German Research Center for Geosciences Potsdam (GFZ) for secondary ion mass spectrometry (SIMS). Otolith sections were embedded into sample mounts with Epofix epoxy resin, along with UWC3 and IAEA603 calcite reference materials (Kozdon et al., 2009; IAEA 2016). The mount was polished to a surface quality of < 10 $\mu m$ surface deviation, which was assessed via white light profilometry. Transects were marked digitally on the otolith sections in a straight line from the core to the distal edge along the longest available axis crossing all visible annuli. Sample mounts were sputter-coated with a 35 nm thick, high-purity gold film and placed in a specially designed high-vacuum storage chamber of the Cameca 1280-HR secondary-ion mass spectrometer. Analyses were performed as point profiles with a step size of ~35 μm along the marked transects, with both reference materials being analyzed after each 10^th^ otolith determination. Results were reported in ‰ relative to Vienna Standard Mean Ocean Water (VSMOW) and converted to Vienna Pee Dee Belemnite scale (VPDB) using the equation by Brand *et al.* (2014). The mounts were transferred to GEOMAR Helmholtz Centre for Ocean Research Kiel for the trace elemental analysis, placed in the sample chamber (LFC – large format cell) of a NewWave UP193fx 193nm excimer laser ablation system (cell gas flow of 0.5 l/min Helium), coupled with a NuInstruments AttoM HR-ICP-MS system. Otoliths were measured using 20 µm spots in the form of line scans at 5 µm/s, 10 Hz repetition rate and fluence of 4.5 J/cm^2^. All samples were preablated using 25 µm spots in line scans of 50 µm/s, 10 Hz, fluence of 4.5 J/cm^2^. The line transects were conducted on top of the SIMS point profile transects, in order to obtain trace element data at the same temporal resolution as the δ^18^O values. The AttoM was operated under hot plasma conditions (normalized Ar index NAI of 26) to provide high matrix- and mass load tolerance during analyses, following Fietzke & Frische (2016). Data were calibrated using a NIST-SRM610 glass reference material measured twice at the beginning and end of each set of otolith samples (7-18) on each mount (**Table S3**). JCp-1 and JCt-1 reference materials were measured alongside the NIST-SRM610 to ensure consistency (**Table S3**). Concentrations of trace elements were reported in elemental concentration ratios in g/g Ca.

**Table S3**. Results of standard materials measured by LA-ICP-MS; displayed as mean (1 SD)

| **Elemental ratio [mg/g]** | **NIST-SRM610**  **N=44** | **JCp-1**  **N=22** | **JCt-1**  **N=22** |
| --- | --- | --- | --- |
| **Na/Ca** | **1221** (27) | **10.45** (0.75) | **10.80** (1.02) |
| **Mg/Ca** | **5.71** (0.12) | **2.45** (0.17) | **0.78** (0.18) |
| **Mn/Ca** | **5.61** (0.10) | **0.0021** (0.0023) | **0.0011** (0.0017) |
| **Sr/Ca** | **6.33** (0.11) | **16.96** (0.92) | **3.59** (0.22) |
| **Ba/Ca** | **5.56** (0.12) | **0.022** (0.007) | **0.012** (0.005) |

## Section C: Statistical analysis of otolith data and ecotype clustering

Sr/Ca is a good proxy for salinity in brackish systems (Kerr *et al.,* 2007; Brown *et al.,* 2009). Therefore, otolith δ^18^O values were tested for intra-otolith correlation with Sr/Ca data from the same location on the otoliths to check for effects of ambient salinity on intraotolith δ^18^O. As δ^18^O values correlated with Sr/Ca (Pearsons R^2^=0.31, p < 0.001), we transformed the δ^18^O values into residuals from a linear regression of δ^18^O on Sr/Ca value, to obtain a relative thermal proxy that was corrected for salinity. To remove noise, trace elemental data were smoothed with a rolling mean of 9 sample points, similar to Hegg and Kennedy (2021). Then, to align trace elemental values with δ^18^O values, data were averaged to the step size of δ^18^O transects (35 µm) via linear interpolation. To correct for the annual seasonal signal in δ^18^O, the δ^18^O residuals were smoothed with a rolling mean of 7 sample points (average number of δ^18^O determinations within a single year ring across all pike in the sample) to obtain an estimate of mean annual thermal niche.

The identified clusters, called clusters A-F (tributary sample, **Fig. S4**) and G-J (lagoon sample, **Fig. S5**) were classified according to the decision tree in **Fig. S6**. Clusters A to C from the tributary sample were identified as freshwater residents, with low Sr/Ca values throughout life. Cluster A showed a relatively cold thermal niche (**Fig. S4 A**), indicated by higher δ18O values, while clusters B and C showed a pronounced ontogenetic thermal shift from warm early life to a colder later life, indicated by shift from low to high δ18O values over the lifetime (**Fig. S4 B** and **C**), likely owing to differences in stream temperature. Cluster D had a clear freshwater origin and experienced intermediate lifelong salinity history indicated by higher Sr/Ca values with frequent oscillations between freshwater and low-saline brackish water, and a strong ontogenetic shift from a warm juvenile niche towards colder water in later life (**Fig. S4 D**). Cluster E was similar to D, but with higher experienced salinity oscillations (**Fig. S4 E**). Clusters D and E were thus identified as anadromous. Cluster F was not easily classified. Most individuals had no clear freshwater origin, and no clear thermal shift similar to the other anadromous clusters was evident (**Fig. S4 F**). Despite having been captured in freshwater tributaries (mainly river Sehrowbach), cluster J was similar in Sr/Ca values to the low saline cluster of the brackish sample (**Fig. S5 G**). We classified this cluster under the term cross-habitat phenotype.

Clusters H-J from the lagoon sample showed a clear brackish water origin signal. Cluster I experienced high salinity and inhabited warm habitats (**Fig. S5 G**), while cluster J experienced similar salinity, but inhabited a colder thermal niche (**Fig. S5 H**). Cluster H showed similar Sr/Ca origin but lower Sr/Ca values in later life compared to clusters I and J, along with a relatively cold adult thermal niche, indicated by high δ18O values after the first few µm on the otoliths (**Fig. S5 I**). Clusters H to J were defined as brackish residents. Cluster G could not easily be defined in terms of origin (**Fig. S5 G**). It showed a lower Sr/Ca signal compared with clusters H-J throughout life, and an origin Sr/Ca signal similar to the origin values of cluster F from the tributary sample (**Fig. S4 F**). Pike of cluster J also showed a pronounced thermal niche shift (**Fig. S5 G**), and were classified as cross-habitat type. Despite our attempt at classification, it should be noted that, when arranged next to each other, the saline histories of all clusters fall into a continuum connecting two endpoints, clusters A and J, which likely resemble the two habitat extremes of full freshwater and full brackish residency. This bears remarkable similarity with behavioral continua described elsewhere in the context of partial migration literature (Cagnacci et al., 2011; Chapman et al., 2011). **Fig. S7 – S10** illustrate typical individuals of the behavioral phenotypes, along with descriptions of the decision process for each.


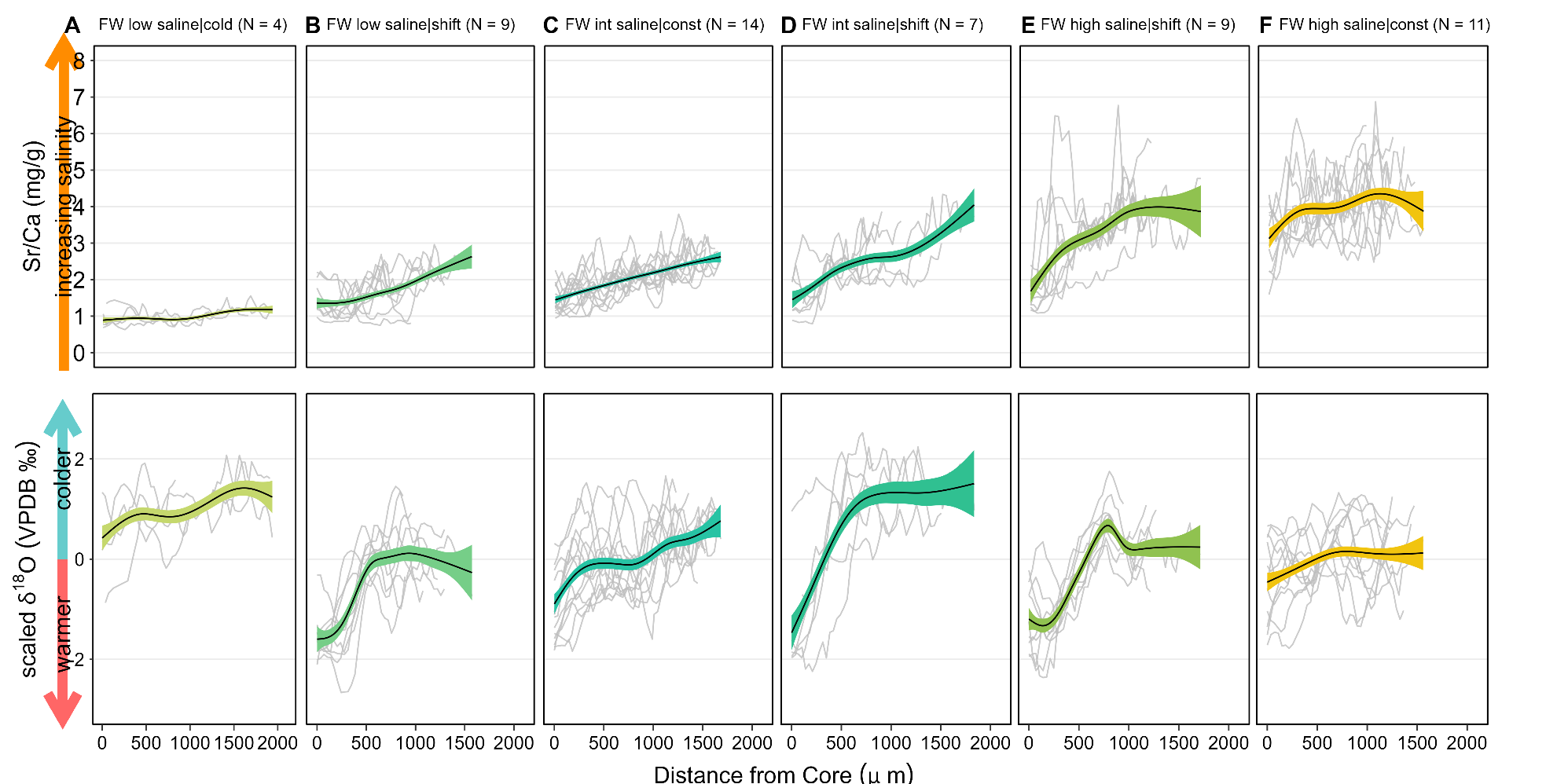


**Fig. S4** Lifelong thermosaline clusters from northern pike (*Esox lucius*) captured in freshwater tributaries of the brackish lagoons around Rügen island, Germany (N = 54) between July 2019 and April 2022, identified by agglomerative hierarchical dynamic time warp clustering. Upper panels show the lifelong Sr/Ca values (mg/g), lower panels show the lifelong δ^18^O residuals (corrected for Sr via linear regression). Clusters A, B and C were assigned to the freshwater resident behavioral phenotype; Clusters D and E were assigned to the anadromous behavioral type; cluster F was assigned to the cross-habitat behavioral type. Cluster centroids are indicated with colored smoothing lines, individual transects are shown in grey lines


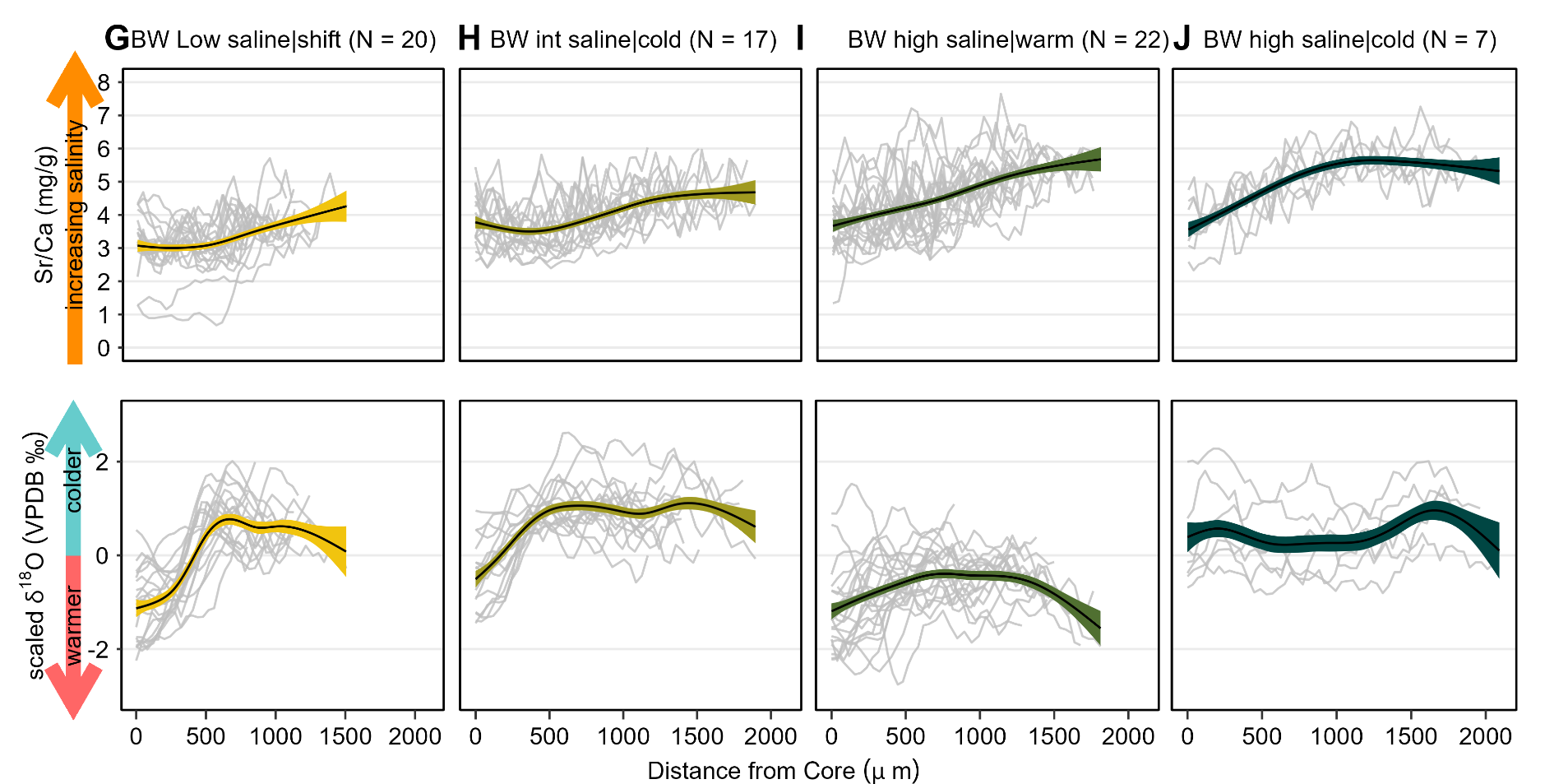


**Fig. S5** Lifelong thermosaline clusters for northern pike (*Esox lucius*) captured from the major brackish lagoon chains WRBC, NRBC and GB (N = 66) around Rügen island, Germany, between November 2019 and January 2021, identified by agglomerative hierarchical dynamic time warp clustering. Upper panels show the lifelong Sr/Ca values (mg/g), lower panels show the lifelong δ^18^O values (corrected for Sr via linear regression). Cluster G was assigned to the cross-habitat phenotype, clusters H, I and J were assigned to the brackish resident phenotype. Two fish from cluster G, one fish from cluster I, and one fish from cluster J were assigned to the anadromous behavioral phenotype due to a clearly distinguishable freshwater origin. Cluster centroids are indicated with colored smoothing lines, individual transects are shown in grey lines


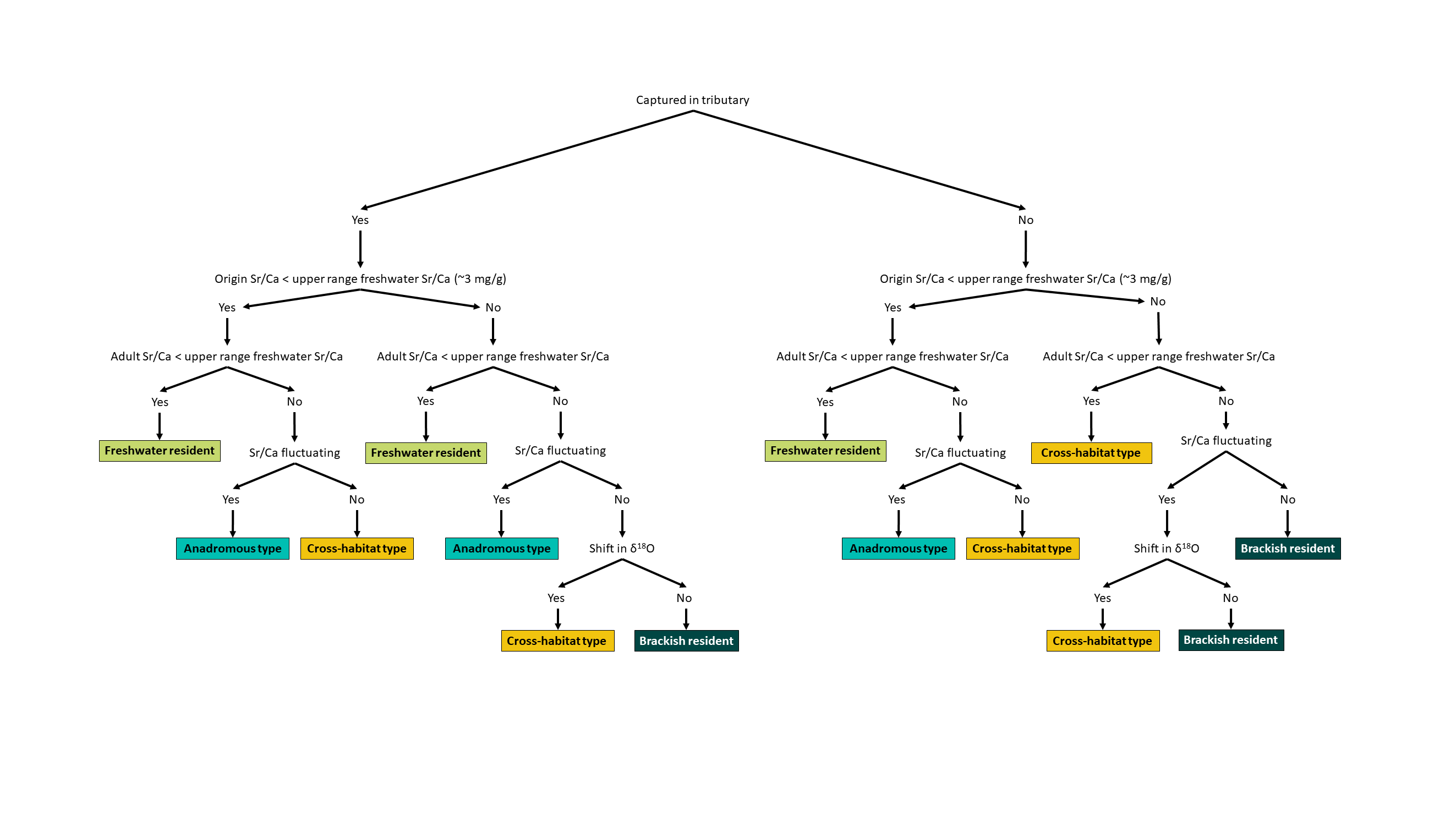


**Fig. S6** Decision tree for categorizing clusters obtained by agglomerative hierarchical dynamic time warp clustering from combined Sr/Ca and δ^18^O transects on northern pike (*Esox lucius*) otoliths (N = 120) into behavioral type categories. Upper freshwater Sr/Ca was ~3 mg/g, calculated by adding 2 x SD of the first 50 µm Sr/Ca transect (after discarding the first 100 µm to remove maternal signal, similar to Möller et al., 2019) to the mean Sr/Ca of the first 50 µm of the tributary sample. A thermal habitat shift in δ^18^O was defined as an increase (or decrease) in more than one permil (roughly equivalent to °C in water temperature) from juvenile (first few µm) to adult phase


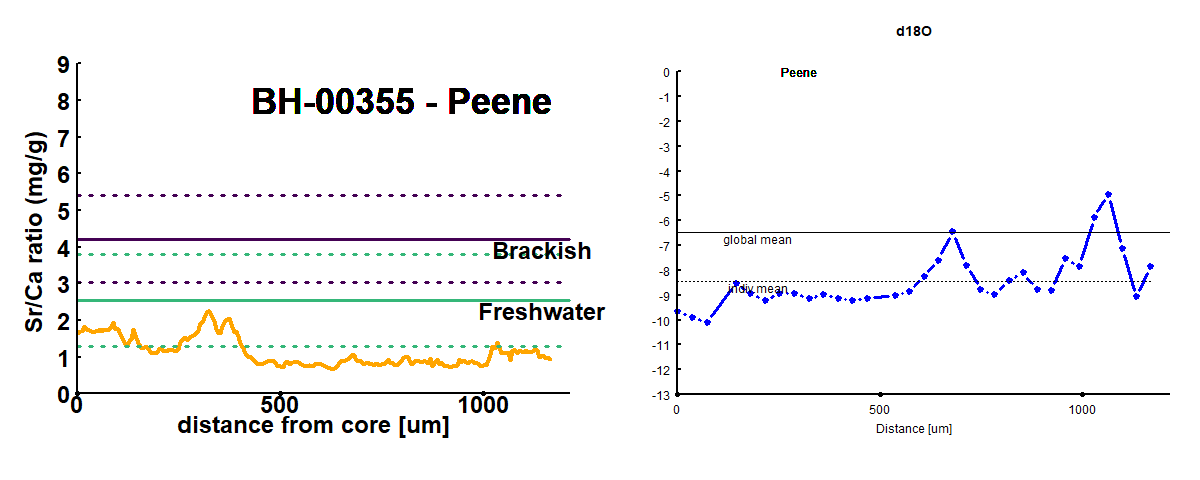


**Fig. S7** Example for a freshwater resident behavioral phenotype, a 55.2 cm (TL) male pike captured in the upper reach of river Peene (**Fig. S1**). Origin (core) and lifelong Sr:Ca values are below the mean freshwater and upper freshwater values (calculated from mean + 2 SD of all freshwater fish caught outside of spawning season, i.e., putative residents). Mean δ^18^O values increase around 1 ‰ over the lifetime of the individual, indicating an ontogenetic shift from juvenile warm to adult cold habitat


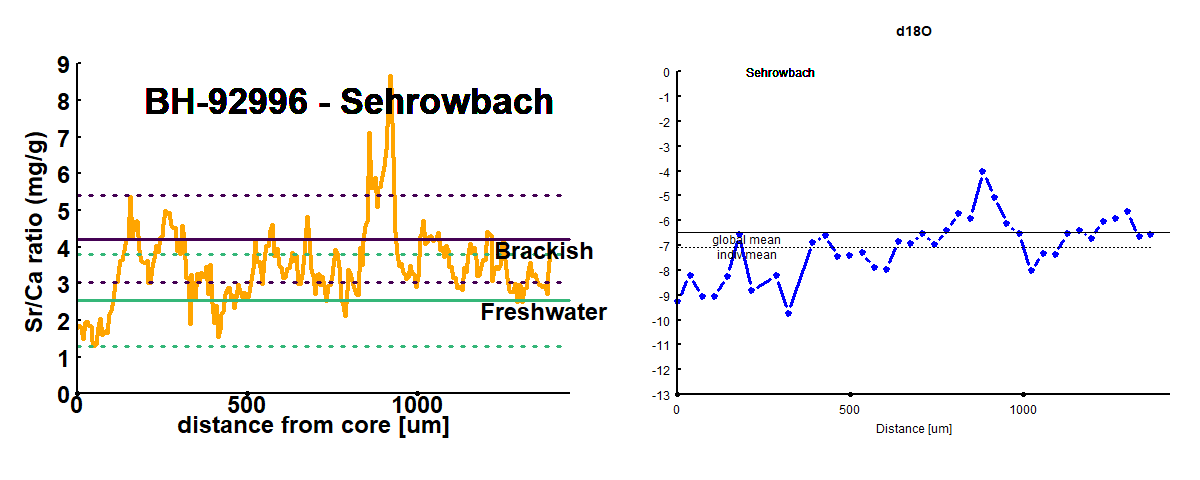


**Fig. S8** Example for a typical anadromous behavioral phenotype, a 66.5 cm (TL) female pike captured in the middle reach of river Sehrowbach (**Fig. S1**). The origin Sr:Ca value clearly indicates the fish was born in freshwater, but Sr:Ca values afterwards increase, indicating a habitat shift to brackish water. However, clear oscillations between habitats were observed in this individual (e.g., at distance from core ~500 µm, 800 µm, 1000 µm). A marked increase in mean δ^18^O from early to later life indicates the ontogenetic habitat shift from a warm nursery habitat to a colder adult habitat


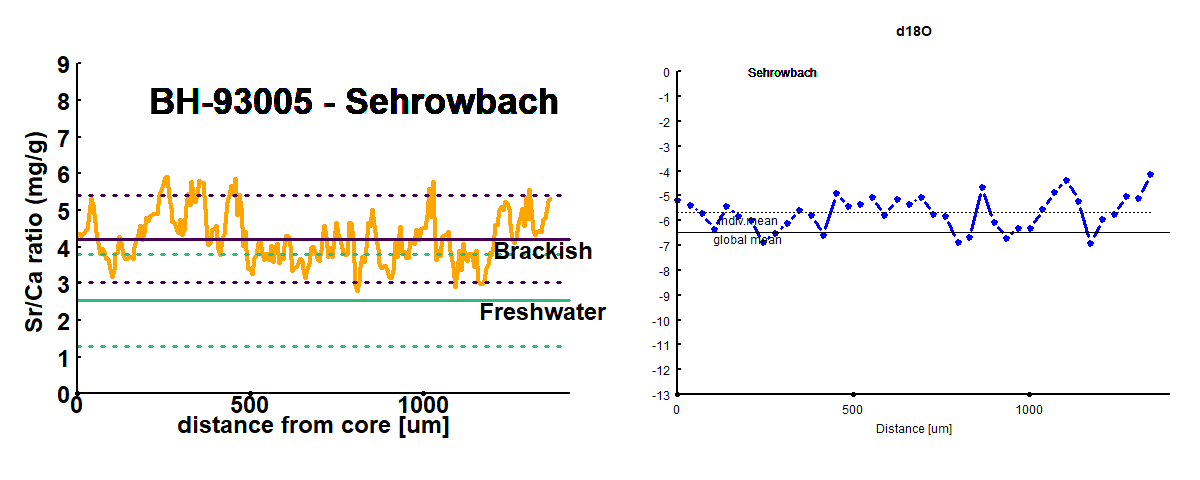


**Fig. S9** Example for a typical cross-habitat behavioral phenotype, a 76.5 cm (TL) female pike captured in the lower reach of river Sehrowbach during peak spawning season. Despite this, origin Sr:Ca values indicate the fish was born in brackish water. The lifelong Sr:Ca values show no clear freshwater oscillations, however, values are mostly below the mean brackish values and within the upper freshwater values for long periods of time. δ^18^O values do not show the marked ontogenetic shift as observed in anadromous behavioral phenotypes


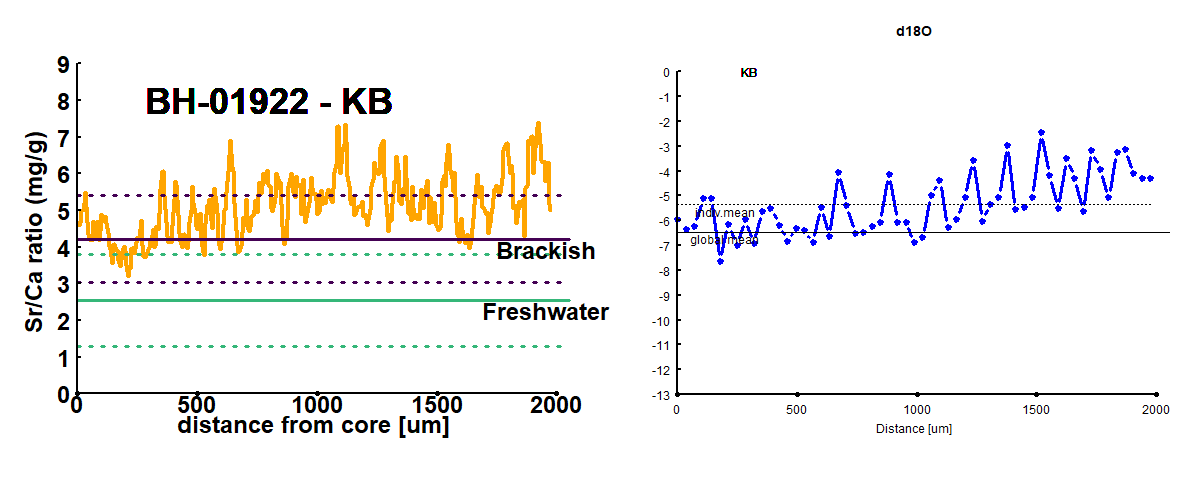


**Fig. S10** Typical brackish resident behavioral phenotype, a 126.2 cm (TL) female caught in Schaproder Bodden in the WRBC (**Fig. S1**). Origin Sr:Ca values clearly indicate the fish was born in brackish water, lifelong Sr:Ca values are around or above mean brackish water values (calculated from all fish caught in brackish water ± 2 SD) for the entire life, and mean δ^18^O values are stable across the entire life, indicating no larger ontogenetic habitat shifts (i.e., one habitat throughout the entire life history)

## Section D: Genetic analyses

## SNP panel development

The genetic distances described by Roser et al. (2023) among pooled samples from 11 sites differing in their salinity or with respect to migratory status of sampled pike strongly suggested that genetically divergent lineages occur in the study area. Here we developed a marker panel of SNP (single nucleotide polymorphism) loci that best captures the underlying populations structure with the goal to genotype and assign individual pike to divergent populations. Genomic regions with signatures of high between population divergence were explored to identify the functions of genes that might be directly involved in population divergence following the ideas of Wu (2001) who outlined how adaptive evolution causes elevated levels of genomic divergence for adaptive genetic traits. To develop a SNP panel with loci to discriminate brackish-water, freshwater and anadromous pike, we screened previously published (Roser et al. 2023) whole genome (pool-seq) data from 11 locations, including mesohaline brackish-water (Barther, Breeger, Kubitzer, Schaproder, Greifswalder, Strelasund, Wieker, Großer Jasmunder, Kleiner Jasmunder Bodden), possibly resident/anadromous freshwater (rivers Barthe, Peene, Recknitz), oligohaline brackish environments (Peenestrom, Saaler Bodden) and putative anadromous populations (Sehrowbach, Duwenbeek, Neuendorfer Hechtsgraben, Ziese River, Körkwitzer Bach). Sequence data were quality trimmed with trimmomatic (Bolger et al. 2014) and mapped against an annotated reference genome (GCF_011004845.1, downloaded from NCBI) using NextGenMap (Sedlazeck et al. 2013). After converting the resulting sam files into a binary (bam) format, files were combined into a mpileup file and simplified into a sync-format, which was filtered for a minimum count of five of the minor alleles, a minimum allele frequency of 10% of the minor alleles, a minimum coverage of 20 and a maximum coverage corresponding to the average plus two times the standard deviation of the pool (see also Roser et al. 2023). The filtered sync-file was screened for SNPs with the highest allele frequency differentials among brackish-water, freshwater and anadromous groups, and for the presence of diagnostic SNPs for single anadromous streams (Sehrowbach, Neuendorfer Hechtsgraben, Ziese River) using custom perl scripts. Highly differentiated SNPs were chosen if the difference among groups exceeded the range of allele frequency differentials within groups. After visual inspections for mapping artifacts using IGV (https://software.broadinstitute.org/software/igv), we developed primers for 33 loci using the software Primer3 (https://primer3.ut.ee/), aiming for 50-70 bp sequence length surrounding the target SNP, a Tm of 52-57°C and primer sizes of 17-22bp without SNPs in the primer regions (see **Table S4**). The gene content and functions associated with the marker panel was identified through the genome annotation available at NCBI (GCF_011004845.1_fEsoLuc1.pri_genomic.gtf) and a literature screen for associated gene functions.

**Sampling, DNA extraction, sequencing**

Extraction of DNA from fin-clip tissues stored in ethanol followed a standard phenol-chloroform protocol (Sambrook et al. 2001). We employed the genotyping-in-thousands by sequencing (GT-seq) method (Campbell et al. 2015) to determine individual genotypes for our SNP panel of 33 loci with the help of Illumina sequencing (150 bp, paired-end, sequenced at CeGaT, Tübingen, Germany). GT-seq allows cost-effective genotyping of thousands of individuals in a single Illumina run, using two rounds of PCRs that attach unique barcode adapters to each individual (Campbell et al. 2015).

**Analyses**

Raw Illumina sequence reads were quality trimmed and cleaned from adapter sequences using the software trimmomatic (Bolger et al. 2014) (parameters: ILLUMINACLIP:adapter.fasta:2:30:10 MINLEN:50 MAXINFO:40:0.2). Trimmed sequences were then mapped to a fasta formatted file containing sequences of the target loci with NextGenMap (Sedlazeck et al. 2013). Resulting sam files were transformed into a binary (bam) file format in samtools (Li et al. 2009). Next, we used bcftools (Li 2011) to build a vcf file for each locus and filter for a minimum coverage of 10 and against indels closer than 5bp to the target SNP. Locus-specific vcf files were then concatenated using vcftools (Danecek et al. 2011). We kept only target SNPs and loci or individuals with less than 20% missing data. For subsequent analyses in STRUCTURE (Pritchard et al. 2000), we transformed the vcf file into a compatible format using the Stacks software (Catchen et al. 2013). We used an admixture model in STRUCTURE to perform 5 replicate runs (burn-in: 50.000 steps, MCMC: 100.000 steps) and used STRUCTURE HARVESTER (Earl & von Holdt 2011) to find the number of genetic clusters that fits the data best, based on Evanno's delta K method (Evanno *et al.,* 2005). This resulted in k = 4 clusters as ideal solution (**Fig. S9**). We derived assignment probabilities to each of 4 genetic clusters for all 101 pike with isotope data available from the STRUCTURE data file.

**Table S4** Information to the 33 loci used as SNP panel for assigning northern pike (*Esox lucius*, N = 101) captured between July 2019 and April 2022 in the brackish lagoons and several freshwater tributaries around Rügen island in Germany to four genetic clusters

| **Locus and gene name** | **Position in the reference genome** (GCF_011004845.1_fEsoLuc1.pri) | **Primer sequences**  (F: forward; R: reverse) | **Diagnostic**  **assignment** |
| --- | --- | --- | --- |
| Snx19  (Sorting Nexin 19) | NC_047569: 24,347,444 | F: TCAGAGGCTTGAACAGTATC  R: TGGTTAGAGGTCTTAGAGGA | Fresh- vs. brackishwater |
| LOC109615708 | NC_047572: 13,494,631 | F: AAAATCATGTTGGCCTGGA  R: CTTTTCTGTTTAAGGCGACA | Anadromous vs. Freshwater |
| Neurexin  (Neurexin1) | NC_047573 : 7,769,557 | F: AGGTAGCACTAACTCGGG  R: TCGAGCAAATCTAGGAATAAAC | NHG vs. all others |
| Shisa9a  (Shisa Family Member 9) | NC_047573: 17,599,435 | F: GTGTAGAACAGCCACATAAT  R: TGTCTGAACATACTGGGTAG | Anadromous vs. Brackishwater |
| Near_si:zfos-911d5.4 | NC_047574: 6,871,283 | F: GTTAACCAACCCATTCTTTG  R: GGTGACTGTCAACGATATTT | Anadromous vs. Freshwater |
| Intergenic2 | NC_047574: 3,941,419 | F: TTGAATCTCCACACTGTACA  R: AGCAATGTCAATAATCACGG | Fresh- vs. brackishwater |
| Kctd16b  (Potassium Channel Tetramerization Domain Containing 16) | NC_047575: 44,778,492 | F: TTAGACTTCGTAGGGCATAC  R: ATTGACCATCAGTGACACC | Sehrowbach vs. all others |
| Near_ptprfa  (Close to Protein Tyrosine Phosphatase Receptor Type F) | NC_047576: 24,504,780 | F: AATGCACAAAATCTTCTGGA  R: TGCCAAGAATAAAGTTCAGC | Sehrowbach vs. all others |
| Wipf3  (WAS/WASL Interacting Protein Family Member 3) | NC_047578: 635,371 | F: ACATTCATGATTGTAGGCTG  R: GAATTTACTATATTGCTGGAGG | Ziese vs. all others |
| Intergenic1 | NC_047578: 17,384,430 | F: GAGATGGGTGAGTACAGATT  R: CTCGTCACGCTAAGGAG | NHG vs. all others |
| Teashirt  (Teashirt Zinc Finger Homeobox 1) | NC_047578: 17,423,433 | F: GATCAAGTGAACGGGGAT  R: CCAATAAGGGGAATAAGCTT | NHG vs. all others |
| LOC105006983 | NC_047578: 18,969,415 | F: GTGACGGGTCCTTTTGA  R: GAAACCACCATGTCAACTAG | Ziese vs. all others |
| LOC114840414 | NC_047579: 31,539,939 | F: GATAAAAGGATGTGCTTGCC  R: GGGCTGTAAATATGCTTTTC | NHG vs. all others |
| Stat5a  (Signal Transducer and Activator of Transcription 5A) | NC_047579: 42,559,309 | F: AGTCGATCGTATCCCTAGTA  R: ACATACAAATATCGCTAAGCA | Fresh- vs. brackishwater |
| Mitf  (Melanocyte Inducing Transcription Factor) | NC_047580: 23,715,258 | F: GCACTACTTTTATTGGTCCA  R: TCAATTTCTAGCTTTTCCTCAG | Fresh- vs. brackishwater |
| Git2a  (G Protein-Coupled Receptor Kinase Interacting ArfGAP 2) | NC_047581: 26,987,161 | F: ACGCTGCACAGGAGATG  R: AGGAGTGCAGAAATTGAGAT | Ziese vs. all others |
| lrp2a  (Low density lipoprotein-related protein 2) | NC_047584: 17,665,030 | F: TGACCGCTTCAAATGTGA  R: CGACAAGATATCTCGTCAGT | NHG vs. all others |
| Map2  (Microtubule-Associated Protein 2) | NC_047584: 21,734,812 | F: TGATACACAACCATCCTCTA  R: ACATGACACTAATAGAATGACA | NHG vs. all others |
| Csmd3  (CUB and sushi domain-containing protein 3) | NC_047584: 29,907,226 | F: GATATCAGGGAAGTGCTGT  R: AATGAGACAATCCAATCCTG | NHG vs. all others |
| Alpl  (Alkaline Phosphatase, Biomineralization Associated) | NC_047585: 22,846,325 | F: GGGCATGGCAAATTGAATTA  R: GGTTTCTGTAAGGTAAAGTCT | Fresh- vs. brackishwater |
| Tspan8  (Tetraspanin 8) | NC_047586: 3,400,630 | F: AAAACGGAACCATAAGTGTT  R: TTCTCCCTGGAACCAAAA | Sehrowbach vs. all others |
| Plcb1  (Phospholipase C Beta 1) | NC_047586: 21,294,650 | F: CTCTGAAAGCATGGTCCA  R: ACTCTGGTACTTTATGATAGCT | Fresh- vs. brackishwater |
| Near_gzf1  (Close to GDNF Inducible Zinc Finger Protein 1) | NC_047586: 21,587,982 | F: TCGACTCTGTCCAACTTG  R: ATGAGATGGAAGTGAGAGTC | Sehrowbach vs. all others |
| Strap  (Serine/Threonine Kinase Receptor Associated Protein) | NC_047587: 17,636,093 | F: ATTCCTTGTCTGGTGCAG  R: CCCAGGTTTGACAATTATCA | Anadromous vs. Brackishwater |
| Rhcg  (Rh Family C Glycoprotein) | NC_047587: 17,773,190 | F: TCTTCACATATTACAGGCCA  R: GTAATAAGCCCCGAACGT | Fresh- vs. brackishwater |
| Zp3  (Zona Pellucida Glycoprotein 3) | NC_047587: 20,109,358 | F: AATGTAGTCGTTGCAGCTAT  R: TTTGAGAACAGATATGGCAC | Fresh- vs. brackishwater |
| LOC105021879 | NC_047588: 13,043,665 | F: CAACATTAACCAACGTATTCA  R: GGGGCCTTTAAAGACAAATT | Sehrowbach vs. all others |
| Nrn1A1  (Neuritin 1) | NC_047589: 10,560,642 | F: GCATAACCATTCCTAAACACA  R: AAGACCAATAGCCTACTAGT | Fresh- vs. brackishwater |
| Tubb6  (Tubulin Beta 6 Class V) | NC_047589: 10,863,692 | F: TTGCCAACAATCAGGTAAC  R: ACAATCATCCAGTCCTAAGG | Anadromous vs. Brackishwater |
| Nfkbiz  (NFKB Inhibitor Zeta) | NC_047590: 15,488,112 | F: TTCAAACCAGTAGACCATGA  R: ACACCATTTATCTGCGATTG | Ziese vs. all others |
| Ergic2  (ERGIC And Golgi 2) | NC_047591: 13,545,967 | F: GTCTTCCAGATGCTTATGTG  R: TATTCCTGCTGTACATGGTT | Ziese vs. all others |
| Aloxe3  (Arachidonate Lipoxygenase 3) | NC_047593: 12,878,238 | F: GCAGGTCTAGCTCTATCAG  R: GTCCATTTGAGGTTAGTTGC | Anadromous vs. Brackishwater |
| Slam  (Signaling Lymphocytic Activation Molecule Family Member 1) | NC_047593: 6,193,260 | F: GCTAAACAGGGAAGCCTT  R: GGTGATGGTACTATTCTCAAC | Sehrowbach vs. all others |

**Fixed cluster assignments**

Based on a threshold of > 0.7 assignment probability , which presented the best compromise between retaining fish in the sample (avoiding too many “unassigned” individuals) and conservative assignment to genotypes, we assigned a fixed genotype to each individual fish in our analysis. If no genotype reached an assignment probability of > 0.7, it was classified as “unassigned”. Based on the discrete genotypes, we tested the frequency of genotypes in response to the behavioral types observed through otolith chemistry using a χ^2^-test. The observed phenotypes corresponded significantly with the assigned genotypes (χ^2^ = 81.96, df = 9, p < 0.0001). Similar to the analysis of assignment probabilities, the putatively anadromous and freshwater genotypes consisted of a mixture of behaviorally anadromous and freshwater resident individuals (**Fig. S11**), whereas the cross-habitat behavioral phenotype occurred only in anadromous and brackish genotypes, with the largest proportion being unassigned, indicating the mixed and less fixed genetic composition found for this phenotype. Genotypes varied significantly in their frequency by location (χ^2^ = 76.70, df = 9, p < 0.0001)(**Fig. S11, S12**).
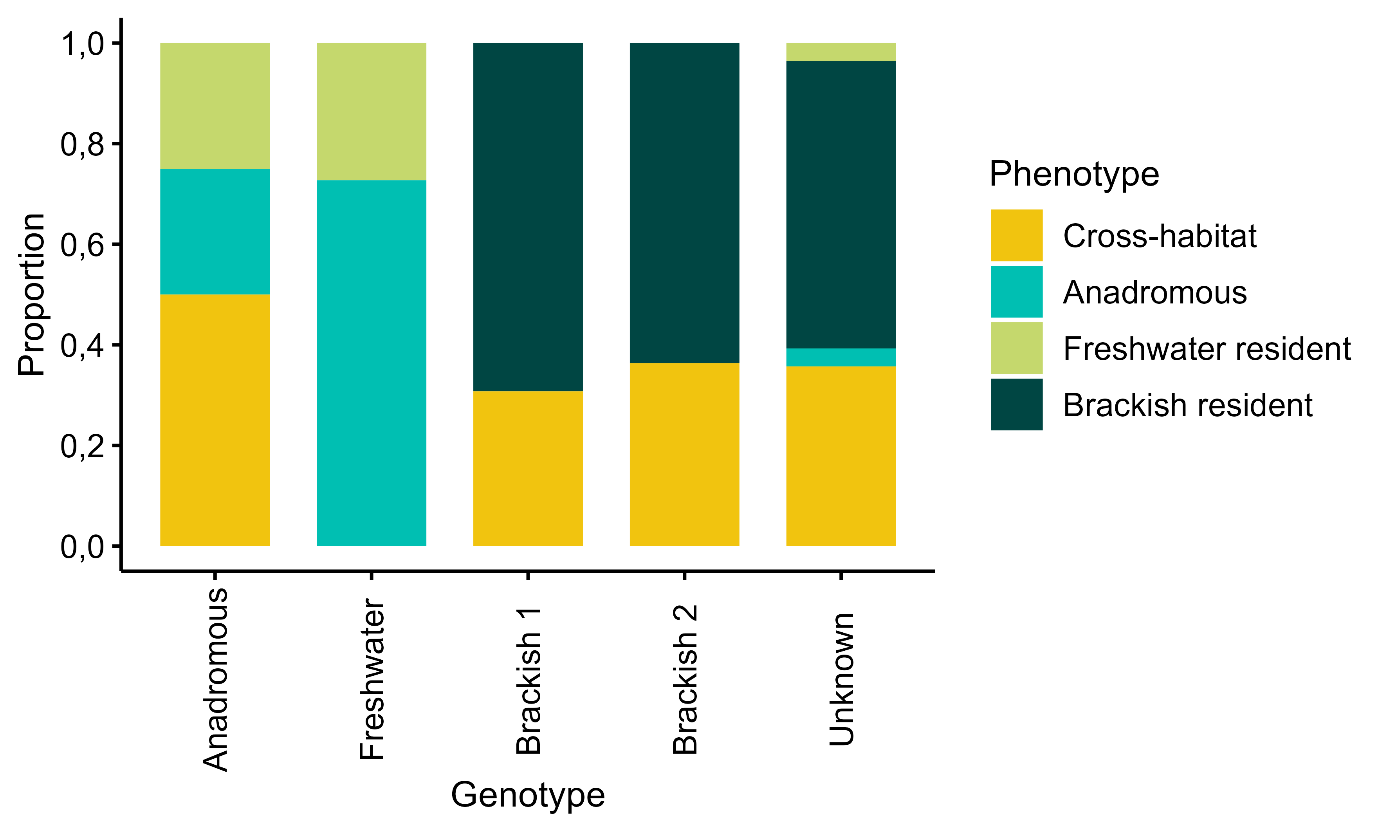


**Fig. S11** Assignment of four behavioral phenotypes to four genotypes for northern pike (*Esox lucius*, N = 101) captured between July 2019 and April 2022 in the brackish lagoons and freshwater tributaries around Rügen island in Germany. Genotypes were differentiated at the individual level based on a threshold assignment probability of 0.7. Individuals for which no assignment probability for one of the genotypes was greater than 0.7 were assigned “unknown”


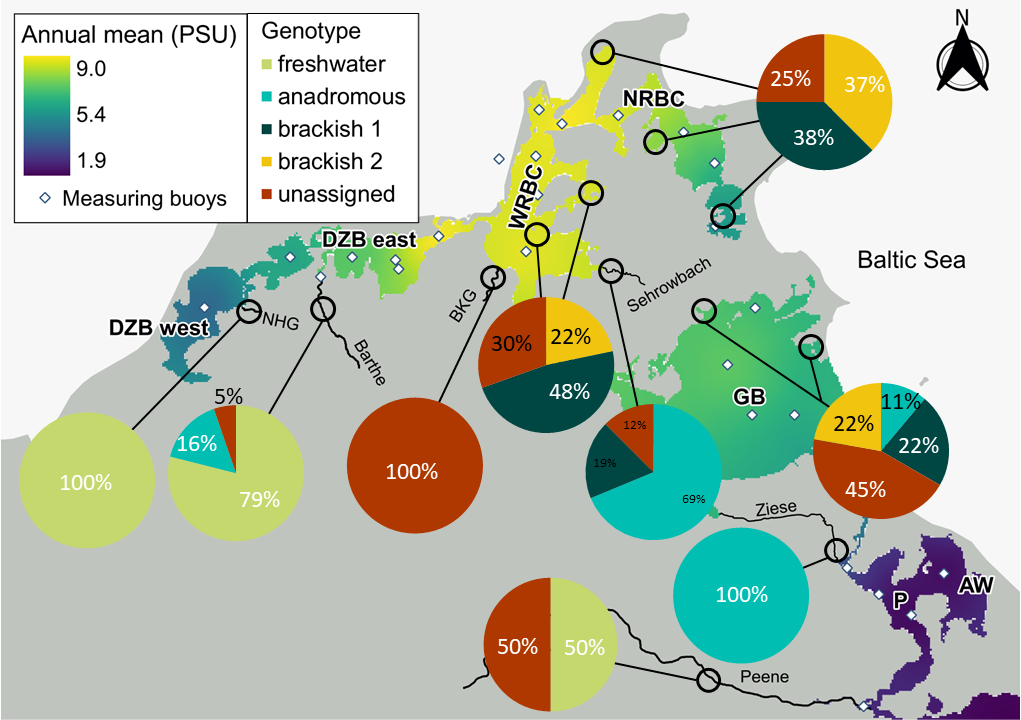


**Fig. S12** Spatial distribution of three putative genotypes assigned to northern pike (*Esox lucius*, N = 101) sampled between July 2019 and April 2022 in brackish lagoons and freshwater tributaries around Rügen island. NRBC: North Rügen Bodden chain, WRBC: West Rügen Bodden chain, GB: Greifswalder, NHG: Neuendorfer Hechtsgraben, BKG: Badendycksgraben.

## Section E: Linear mixed modelling of otolith increments

Genotype showed no significant effect on age-dependent growth of pike in our study (LLR = 12.45, p = 0.26), and was dropped from the final model (**Fig. S13**). This was likely due to the mixed distribution of the fast-growing cross-habitat behavioral phenotype across the anadromous, brackish 1, brackish 2 and unassigned categories, which may have masked any growth differences between these genotypes.


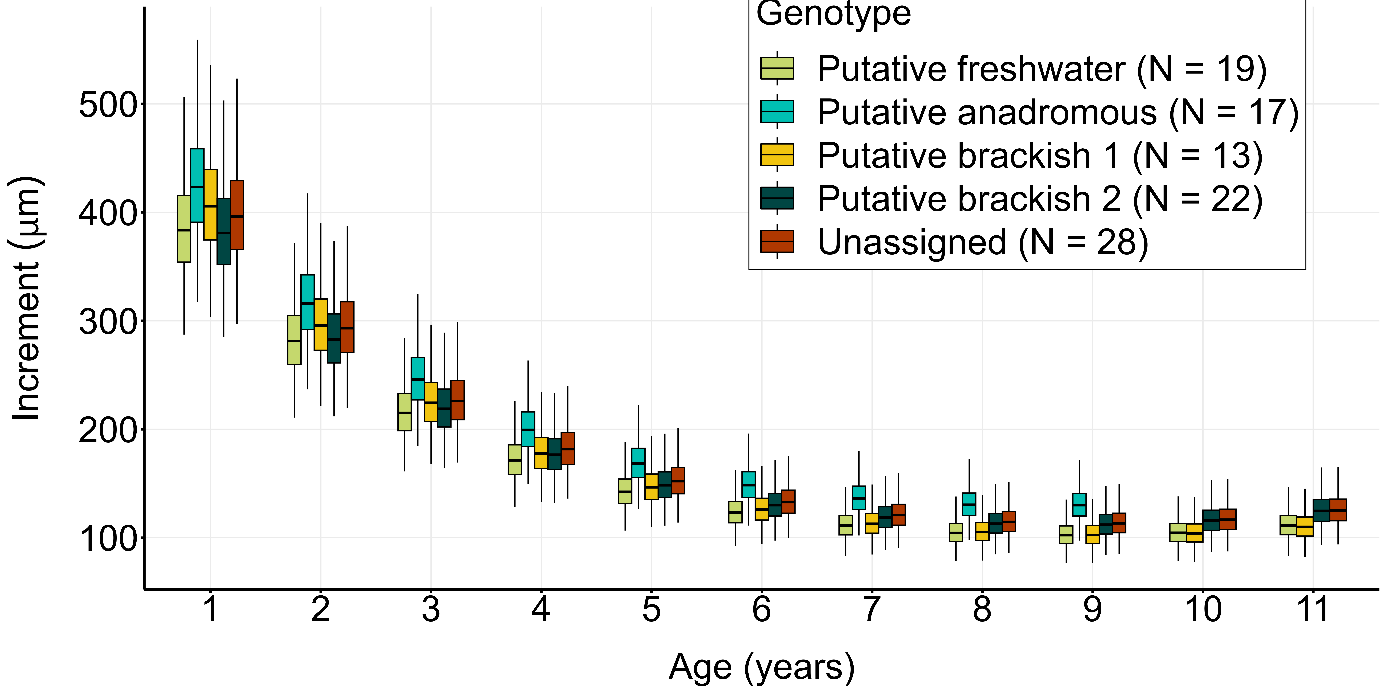


**Fig. S13** Predicted otolith increments compared for four genotypes and unassigned individuals calculated from growth data of northern pike (*Esox lucius*, N = 101) sampled between July 2019 and April 2022 in the lagoons and several tributaries around Rügen island in Germany.

## Section F: Hierarchical von Bertalanffy growth modelling

As expected from the age-specific growth modelling, no differences in lifelong growth were observed for genotype-specific von Bertalanffy parameter estimates (**Table S5**, **Fig. S14,** ). Similarly, we detected no difference in lifelong growth trajectories between ecotypes (**Table S6**, **Fig. S15**).

**Table S5** Genotype-specific von Bertalanffy parameters of northern pike (*Esox lucius*, N = 101) sampled between July 2019 and April 2022 from brackish lagoons and freshwater tributaries around Rügen island. Parameter values are given in interquartile range from the 2.5% to the 97.5% credible parameter space. Values in brackets denote the median parameter estimate

| **Genotype** | **L_∞_** | **k** | **t_0_** |
| --- | --- | --- | --- |
| Putative freshwater | 2.62 - 3.21 (2.88) | 0.10 - 0.13 (0.12) | -0.82 - -0.56 (-0.69) |
| Putative anadromous | 2.70 - 3.46 (3.04) | 0.11 - 0.14 (0.12) | -0.80 - -0.49 (-0.67) |
| Putative brackish | 2.52 - 2.92 (2.70) | 0.10 - 0.12 (0.11) | -0.86 - -0.68 (-0.76) |
| Unassigned | 2.60 - 3.25 (2.89) | 0.10 - 0.13 (0.12) | -0.84 - -0.57 (-0.70) |


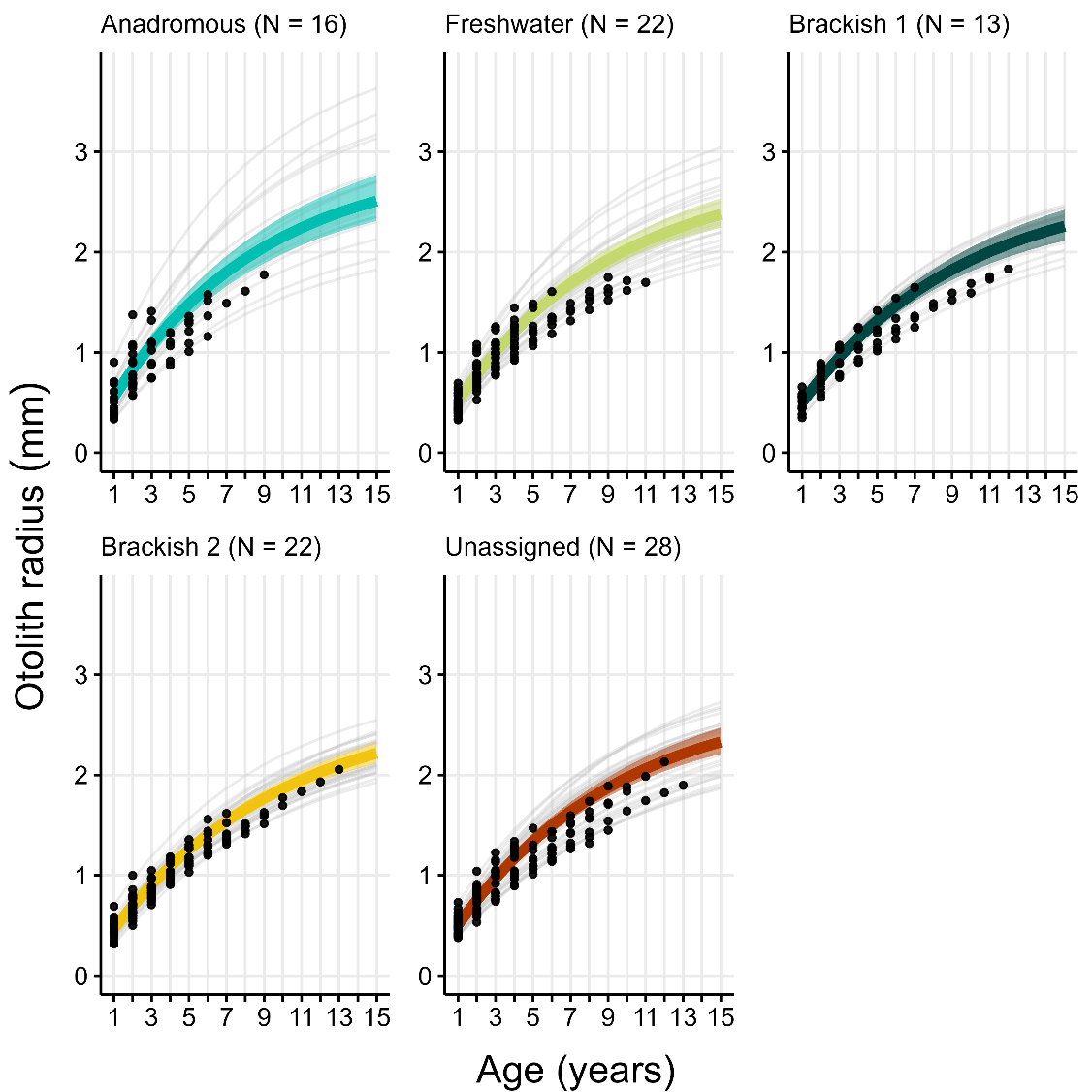


**Fig. S14** Von Bertalanffy growth curves of four assigned genotypes described in northern pike (*Esox lucius*, N = 101) sampled between July 2019 and April 2022 in brackish lagoons and freshwater tributaries around Rügen island, Germany. Grey lines represent individual-level growth curves. Colored lines represent mean radius-at-age of phenotypes, with shaded areas indicating 95% credible intervals.

Table S6 Ecotype-specific von Bertalanffy parameters of northern pike (*Esox lucius*, N = 101) sampled between July 2019 and April 2022 from brackish lagoons and freshwater tributaries around Rügen island. Parameter values are given in interquartile range from the 2.5% to the 97.5% credible parameter space. Values in brackets denote the median parameter estimate

| **Ecotype** | **L_∞_** | **k** | **t_0_** |
| --- | --- | --- | --- |
| Freshwater/anadromous | 2.67 - 3.13 (2.88) | 0.11 - 0.13 (0.12) | -0.75 - -0.55 (-0.65) |
| Cross-habitat | 2.69 - 3.31 (2.96) | 0.10 - 0.13 (0.12) | -0.79 - -0.58 (-0.68) |
| Brackish | 2.43 - 2.76 (2.58) | 0.11 - 0.13 (0.12) | -0.84 - -0.65 (-0.73) |


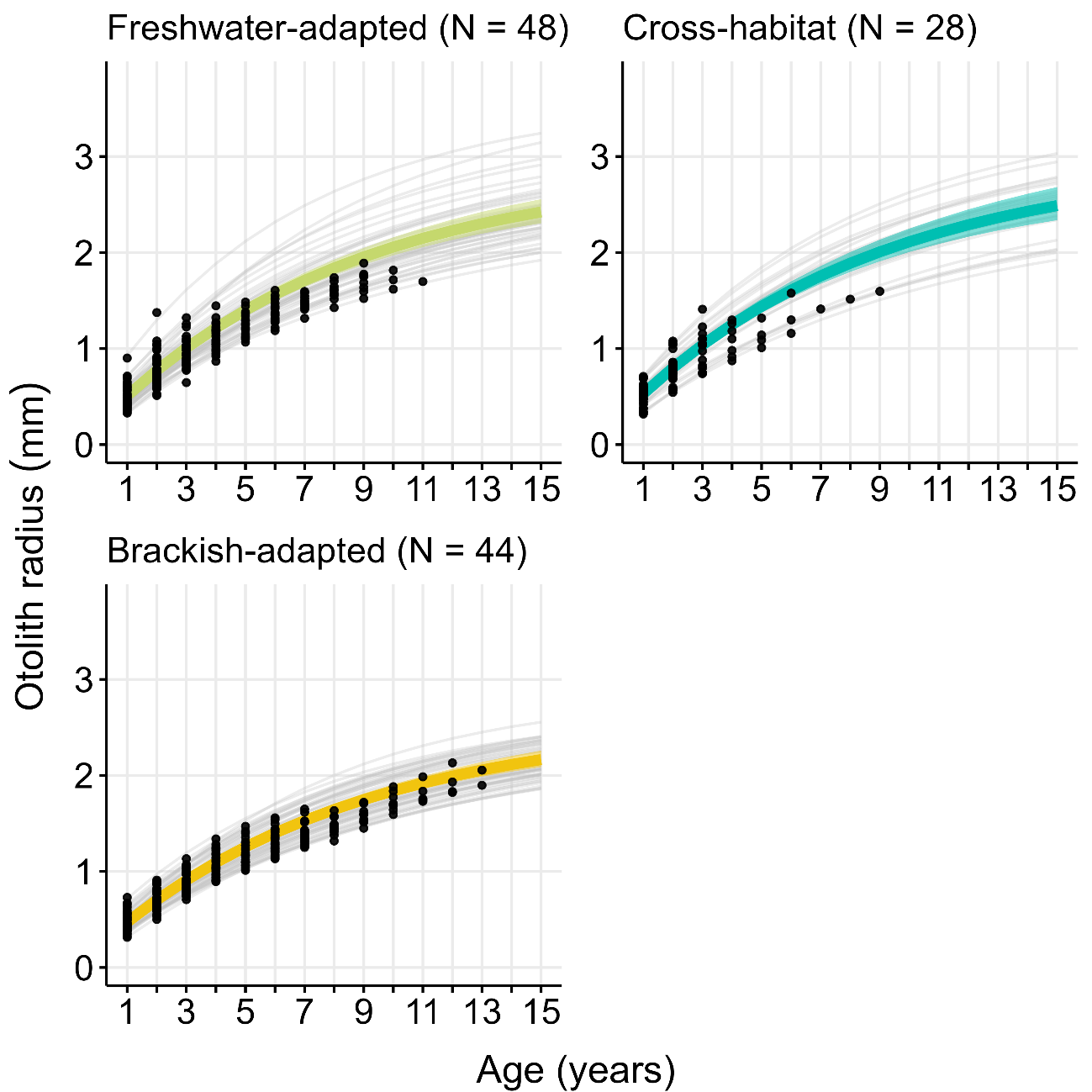


**Fig. S15** Von Bertalanffy growth curves of four identified ecotypes described in northern pike (*Esox lucius*, N = 101) sampled between July 2019 and April 2022 in lagoons and tributaries around Rügen island, Germany. Grey lines represent individual-level growth curves. Colored lines represent mean radius-at-age of phenotypes, with shaded areas indicating 95% credible intervals.


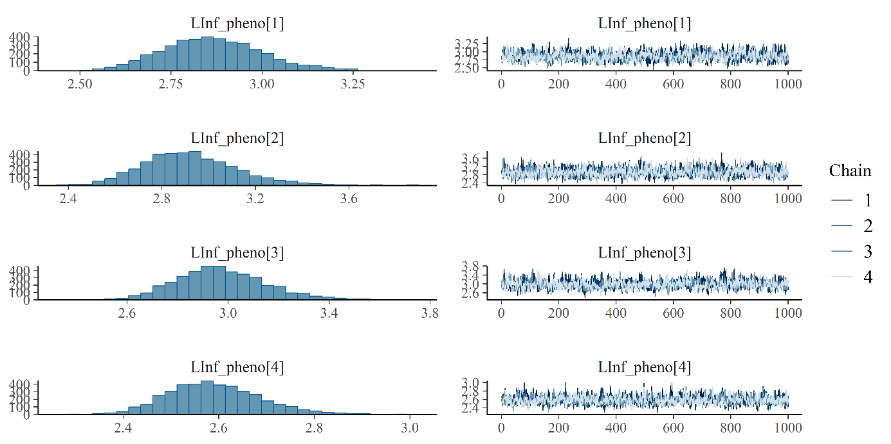


**Fig. S16** Example of histogram and trace plots for the parameter L_∞_ used to evaluate model performance and mixing of chains in the three-level hierarchical von Bertalanffy modelling of phenotype growth curves on otolith increment data of northern pike (*Esox lucius*, N = 101) captured between July 2019 and April 2022 in the brackish lagoons and several freshwater tributaries around Rügen island in German. Deviations from a normal distribution in the histograms or separated chains in the trace plots would indicate sampling problems.


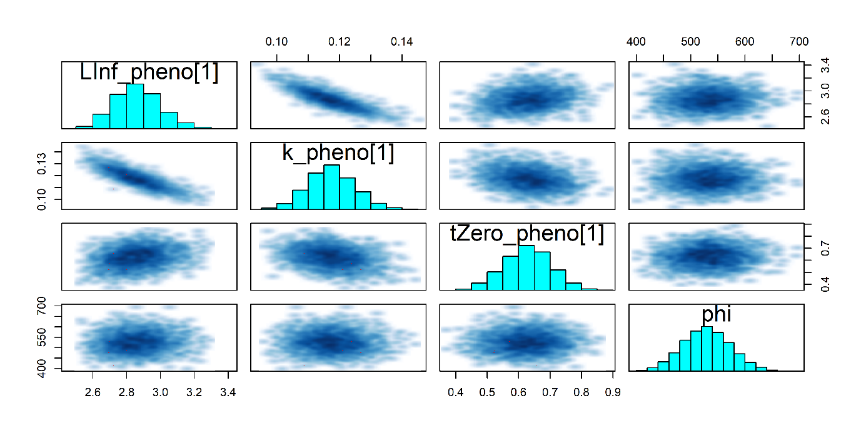


**Fig. S17** Example of a pairs plot evaluating parameter autocorrelation of the von Bertalanffy parameters for behavioral phenotype 1 (freshwater resident). Red points indicate divergent transitions on otolith increment data of northern pike (*Esox lucius*, N = 101) captured between July 2019 and April 2022 in the brackish lagoons and several freshwater tributaries around Rügen island in German. Deviations from normal distributions in the histograms, or patterns in the scatterplots would indicate autocorrelation issues of the model. The weak autocorrelation indicated between L_∞_ and k was expected and is inherent in von Bertalanffy growth curves (Xiao, 1994)

## References

Bolger, A.M., Lohse, M. & Usadel, B. (2014) Trimmomatic: a flexible trimmer for Illumina sequence data. Bioinformatics 30:2114-2120. <https://doi.org/10.1093/bioinformatics/btu170>

Brand, W.A., Coplen, T.B., Vogl, J., Rosner, M. & Prohaska, T. (2014) Assessment of international reference materials for isotope-ratio analysis (IUPAC Technical Report). Pure and Applied Chemistry 86:425-467. <https://doi.org/10.1515/pac-2013-1023>

Brown, R.J., Campana, S. & Severin, K.P. (2009) Otolith chemistry analyses indicate that water Sr:Ca is the primary factor influencing otolith Sr:Ca for freshwater and diadromous fish but not for marine fish. Canadian Journal of Fisheries and Aquatic Sciences 66:1790-1808. <https://dx.doi.org/10.1139/F09-112>

Cagnacci, F., Focardi, S., Heurich, M., Stache, A., Hewison, A. J. M., Morellet, N., Kjellander, P., Linell, J.D.C., Mysterud, A., Neteler, M., Delucchi, L., Ossi, F. & Urbano, F. (2011). Partial migration in roe deer: migratory and resident tactics are end points of a behavioural gradient determined by ecological factors. Oikos 120:1790-1802. <https://doi.org/10.1111/j.1600-0706.2011.19441.x>

Campbell, N.R., Harmon, S.A. & Narum, S.R. (2015) Genotyping-in-Thousands by sequencing (GT-seq): A cost-effective SNP genotyping method based on custom amplicon sequencing. Molecular and Ecological Resources 15:855-867. <https://doi.org/10.1111/1755-0998.12357>

Catchen, J., Hohenlohe, P.A., Bassham, S., Amores, A. & Cresko, W.A. (2013) Stacks: an analysis tool set for population genomics. Molecular Ecology 22:3124-3140. <https://doi.org/10.1111/mec.12354>

Chapman, B.B., Brönmark, C., Nilsson, J.A. & Hansson, L.A. (2011) The ecology and evolution of partial migration. Oikos 120:1764-1775. <https://doi.org/10.1111/j.1600-0706.2011.20131.x>

Danecek, P., Auton, A., Abecasis, G., Albers, C.A., Banks, E., DePristo, M.A., Handsaker, R.E., Lunter, G., Marth, G.T., Sherry, S.T., McVean, G., Durbin, R. & Genomes Project Analysis, G. (2011) The variant call format and VCFtools. Bioinformatics 27:2156-2158. <https://doi.org/10.1093/bioinformatics/btr330>

Earl, D.A. & VonHoldt, B.M. (2012) STRUCTURE HARVESTER: a website and program for visualizing STRUCTURE output and implementing the Evanno method. Conservation genetics resources 4:359-361. <https://doi.org/10.1007/s12686-011-9548-7>

Evanno, G., Regnaut, S. & Goudet, J. (2005) Detecting the number of clusters of individuals using the software STRUCTURE: a simulation study. Molecular Ecology 14:2611-2620. <https://doi.org/10.1111/j.1365-294X.2005.02553.x>

Fietzke, J. & Frische, M. (2016) Experimental evaluation of elemental behavior during LA-ICP-MS: influences of plasma conditions and limits of plasma robustness. Journal of Analytical Atomic Spectrometry 31:234-244. <https://doi.org/10.1039/C5JA00253B>

Hegg, J.C. & Kennedy, B.P. (2021) Let's do the time warp again: non‐linear time series matching as a tool for sequentially structured data in ecology. Ecosphere 12:e03742. <https://doi.org/10.1002/ecs2.3742>

Hijmans, R. (2022). raster: Geographic Data Analysis and Modeling. R package version 3.5-29, <https://CRAN.R-project.org/package=raster>

International Atomic Energy Agency. *Reference Sheet for IAEA-603*. IAEA, Vienna, 2016-08-28, 7pp. <https://nucleus.iaea.org/rpst/ReferenceProducts/ReferenceMaterials/Stable_Isotopes/13C18and7Li/IAEA-603.htm>

Kerr, L.A., Secor, D.H. & Kraus, R.T. (2007) Stable isotope (δ13C and δ18O) and Sr/Ca composition of otoliths as proxies for environmental salinity experienced by an estuarine fish. Marine Ecology Progress Series 349**:**245-253. <https://dx.doi.org/10.3354/meps07064>

Kozdon, R., Ushikubo, T., Kita, N.T., Spicuzza, M. & Valley, J.W. (2009) Intratest oxygen isotope variability in the planktonic foraminifer *N. pachyderma*: Real vs. apparent vital effects by ion microprobe. Chemical Geology 258:327–337. [https://doi.org/10.1016/j.chemg eo.2008.10.032](https://doi.org/10.1016/j.chemg%20eo.2008.10.032)

Li, H., Handsaker, B., Wysoker, A., Fennell, T., Ruan, J., Homer, N., Marth, G., Abecasis, G., Durbin, R. & Genome Project Data Processing, S. (2009) The sequence Alignment/Map format and SAMtools. Bioinformatics 25:2078-2079. <https://doi.org/10.1093/bioinformatics/btp352>

Li, H.(2011) A statistical framework for SNP calling, mutation discovery, association mapping and population genetical parameter estimation from sequencing data. Bioinformatics 25:2987-2993. <https://doi.org/10.1093/bioinformatics/btr509>

Pritchard, J.K., Stephens, M. & Donnelly, P. (2000) Inference of population structure using multilocus genotype data. Genetics 155:945-959. <https://doi.org/10.1093/genetics/155.2.945>

Roser, P., Dhellemmes, F., Rittweg, T., Möller, S., Winkler, H., Lukyanova, O., Niessner, D., Schütt, J., Kühn, C., Dennenmoser, S., Nolte, A.W., Radinger, J., Koemle, D. & Arlinghaus, R. (2023) Synthesizing historic and current evidence for anadromy in a northern pike (*Esox lucius* L.) meta-population inhabiting brackish lagoons of the southern Baltic Sea, with implications for management. Fisheries Research 260:106560. <https://doi.org/10.1016/j.fishres.2023.106670>

Sambrook, J., Fritsch, E.F. & Maniatis, T. (2001) *Molecular cloning: a laboratory manual (2nd ed.)*. Cold spring harbor laboratory press.

Sedlazeck, F.J., Rescheneder, P. & von Haeseler, A. (2013) NextGenMap: fast and accurate read mapping in highly polymorphic genomes. Bioinformatics 29:2790-2791. <https://doi.org/10.1093/bioinformatics/btt468>

Stachelek, J. (2022). ipdw: spatial interpolation by Inverse Path Distance Weighting. R package version 1.0-0. <https://cran.r-project.org/package=ipdw>

Wu, C.I. (2001) The genetic view of the process of speciation. Journal of Evolutionary Biology 14:851-865. <https://doi.org/10.1046/j.1420-9101.2001.00335.x>

Xiao, Y. (1994). von Bertalanffy Growth Models with Variability in, and Correlation between, K and L∞. Canadian Journal of Fisheries and Aquatic Sciences 51:1585-1590. <https://doi.org/10.1139/f94-157>
